# LncRNA FAISL Inhibits Calpain 2‐Mediated Proteolysis of FAK to Promote Progression and Metastasis of Triple Negative Breast Cancer

**DOI:** 10.1002/advs.202407493

**Published:** 2024-09-17

**Authors:** Yunmei Zhang, Shiyu Wei, Zhengjie Chen, Rui Xu, Shu‐Rong Li, Lili You, Ruiyue Wu, Yin Zhang, Jian‐You Liao, Xiaoding Xu, Erwei Song, Man‐Li Luo

**Affiliations:** ^1^ Guangdong Provincial Key Laboratory of Malignant Tumor Epigenetics and Gene Regulation Guangdong‐Hong Kong Joint Laboratory for RNA Medicine Sun Yat‐Sen Memorial Hospital, Sun Yat‐Sen University Guangzhou 510120 China; ^2^ Breast Tumor Center Sun Yat‐Sen Memorial Hospital, Sun Yat‐Sen University Guangzhou 510120 China; ^3^ Medical Research Center Sun Yat‐Sen Memorial Hospital, Sun Yat‐Sen University Guangzhou 510120 China; ^4^ Department of Immunology School of Basic Medical Sciences Southern Medical University Guangzhou Guangdong 510515 China; ^5^ Department of Endocrinology Sun Yat‐sen Memorial Hospital, Sun Yat‐sen University Guangzhou 510120 China; ^6^ The First Clinical Medical College Lanzhou University Lanzhou 730000 China; ^7^ Department of Cellular and Molecular Diagnostics Center Sun Yat‐Sen Memorial Hospital, Sun Yat‐Sen University Guangzhou 510120 China

**Keywords:** focal adhesion kinase, long non‐coding RNA, nanodelivery system, triple negative breast cancer

## Abstract

Triple negative breast cancer (TNBC) is the most aggressive subtype in breast tumors. When re‐analyzing TCGA breast cancer dataset, we found cell adhesion molecules are highly enriched in differentially expressed genes in TNBC samples, among which Focal Adhesion Kinase (FAK) is most significantly associated with poor survival of TNBC patients. FAK is precisely modulated in the focal adhesion dynamics. To investigate whether lncRNAs regulate FAK signaling, we performed RNA immunoprecipitation sequencing and found FAISL (FAK Interacting and Stabilizing LncRNA) abundantly enriched in FAK‐interacting lncRNAs and frequently overexpressed in TCGA TNBC tissues. FAISL promotes TNBC cell adhesion, cytoskeleton spreading, proliferation, and anchor‐independent survival. FAISL doesn't affect FAK mRNA but positively regulates FAK protein level by blocking Calpain 2‐mediated proteolysis. FAISL interacts with the C‐terminus domain of FAK, whereby masks the binding site of Calpain 2 and prevents FAK cleavage. High level of FAISL correlates with FAK expression in tumor tissues and poor prognosis of TNBC patients. A siRNA delivery system targeting FAISL using reduction‐responsive nanoparticles effectively inhibits tumor growth and metastasis in TNBC mouse models. Together, these findings uncover a lncRNA‐mediated mechanism of regulating FAK proteolysis in the TNBC progression, and highlight the potential of targeting lncRNA FAISL for TNBC treatment.

## Graphical abstract

An FAK interacting and stabilizing lncRNA (FAISL) is identified, which binds FAK C‐terminus and prevents Calpain 2‐mediated FAK proteolysis. FAISL overexpression correlates with poor prognosis of triple negative breast cancer (TNBC) patients and resembles FAK‐induced phenotypes in TNBC cells. Nanoparticle‐meditated FAISL silencing inhibits TNBC growth and metastasis in mouse models, which highlights the potential of targeting FAISL for TNBC treatment.

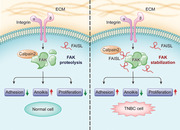

## 1 Introduction

Triple negative breast cancer (TNBC) is the most aggressive subtype in breast tumors, with a higher tendency to metastasize at the early stage. As TNBC lacks expression of hormone receptors (HR) and human epidermal growth factor receptor 2 (HER2), the available biomarkers and targeted therapies are limited. Focal adhesion kinase (FAK), a non‐receptor tyrosine kinase in the cytoplasm, couples with integrins and growth factor receptors to regulate cell adhesion, proliferation, migration, invasion, and metastasis.^[^ ^1^ ^]^ FAK is overexpressed and aberrantly activated in many cancer types, including TNBC, and plays a critical role in tumor progression and metastasis.^[^ ^2^ ^]^

FAK protein is precisely regulated in normal and cancer cells. Upon cell adhesion to the extracellular matrix (ECM), integrin receptors cluster and initiate a cascade where FAK undergoes autophosphorylation at the Y397 residue and is further phosphorylated at additional tyrosine residues by the Src family of kinases to augment the activation. Conversely, FAK is dephosphorylated by phosphatases, such as PTEN and SHP‐2. The regulation of stability of FAK mRNA and protein also contributes to the fine‐tuning of FAK levels. E3 ubiquitin ligases, such as c‐Cbl, facilitate the ubiquitination of FAK, targeting it for proteasomal degradation.^[^ ^3^ ^]^ Importantly, FAK can be proteolyzed during focal adhesion turnover which is mediated by proteases like Calpain‐2 or Caspase‐8.^[^ ^4^ ^]^ The cleavage and subsequent degradation of FAK is a more specific regulation mechanism in the focal adhesion which leads to the disassembly of focal adhesion complexes and the loss of cell adhesion, as well as the interruption of survival signals from the ECM.

Due to the important oncogenic role of FAK, it has long been considered as an ideal target for developing small molecule anticancer drugs. Inhibitors targeting the kinase domain, FERM domain, and FAT domains of FAK have been developed, with different functional mechanisms.^[^ ^5^ ^]^ However, in clinical trials some patients who meet the criteria for FAK targeted therapy do not achieve the expected therapeutic outcomes.^[^ ^6^ ^]^ One reason could be that the regulatory mechanisms of FAK signaling are not fully elucidated, and there may be unknown factors in the pathways that impact treatment efficacy. Therefore, a thorough understanding of the regulatory networks of FAK in TNBC may not only facilitate the development of more specific targets in the FAK pathway but also hold the potential of identifying new biomarkers for targeted therapy.

Long non‐coding RNAs (lncRNAs) have emerged as important modulators of a variety of physiological and pathological processes.^[^ ^7^ ^]^ Growing evidence suggests that lncRNA can regulate multisteps of tumor development through various mechanisms, such as regulating gene transcription, mRNA translation, protein modification, and formation of protein‐protein complexes.^[^ ^7^, ^8^ ^]^ However, whether there are lncRNAs involved in focal adhesion complexes (FAC) and regulate FAK function remains unknown. Moreover, whether lncRNAs can be used as prognostic markers or therapeutic targets in TNBC also requires further research. Here, we aimed to investigate whether lncRNAs interact with FAK and play a role in regulating TNBC progression.

## 2 Result

### 2.1 FAK is Dysregulated and Associated with Poor Prognosis in TNBC

**Figure 1:**
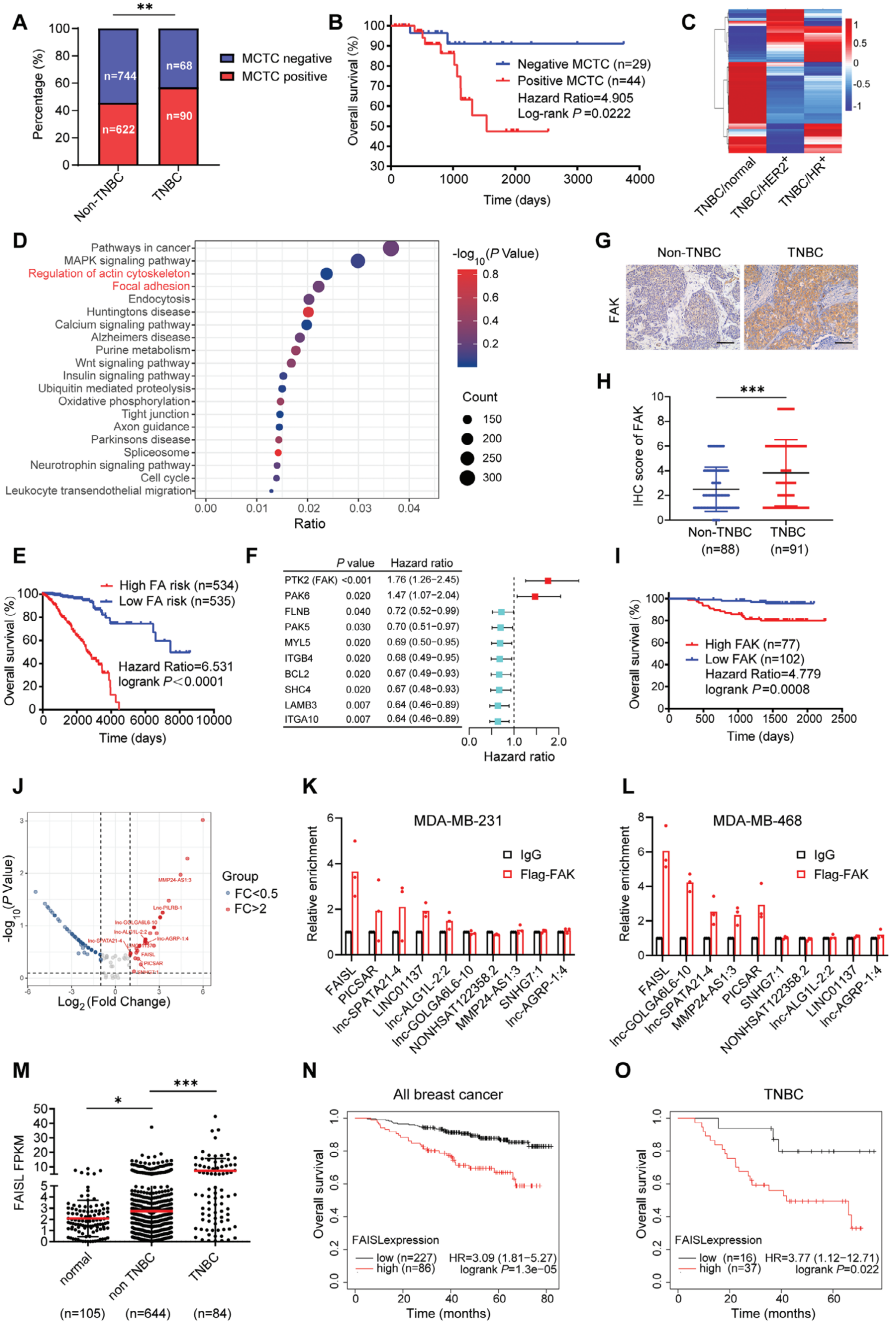
FAISL is a FAK‐associated lncRNA and highly expressed in TNBC. A) The MCTC status in the blood of non‐TNBC and TNBC patients. B) Kaplan‐Meier curve of overall survival of MCTC positive and negative breast cancer patients. C) Heatmap showing the log2 fold change of differentially expressed genes in TNBC, compared with normal, HER2^+^, and HR^+^ subtypes. D) Gene set enrichment analysis of TNBC‐specific differentially expressed genes. E) Polygenic risk analysis and Kaplan‐Meier survival of TCGA breast cancer dataset showing that high risk level of focal adhesion pathway correlates with poor overall survival of breast cancer patients. F) Forest plot showing the univariate survival analysis of the key proteins of the focal adhesion pathway in breast cancer patients. G–H) Representative staining images (G) and statistics (H) of FAK protein level in paraffin‐embedded tumor tissues of non‐TNBC (*n* = 88) and TNBC (*n* = 91) patients, as detected by IHC. IHC signals are detected as yellow/brown staining with nuclei counterstained by hematoxylin. Scale bar, 20 µm. I) Overall survival curve of breast cancer patients with high (*n* = 77) or low FAK (*n* = 102) expression in breast cancer tissues. J) Volcano plot of RIP‐sequencing showing the lncRNAs precipitated by Flag‐tagged FAK in MDA‐MB‐231 cells. FC means fold change of Flag/IgG enrichment. K–L) RIP and RT‐qPCR validation of the top 10 lncRNAs in MDA‐MB‐231 (K) and MDA‐MB‐468 (L) cell lines (*n* = 3). M) Relative FAISL expression in normal breast (adjacent to tumors) (*n* = 105), non‐TNBC (*n* = 644), and TNBC (*n* = 84) tissues from TCGA database. N–O.) Association of FAISL expression with overall survival (OS) in all breast cancer patients (*n* = 313) (N) and TNBC subtype (*n* = 53) (O) from the Kaplan‐Meier Plotter database with RNA sequencing data. Data are presented as the mean ± SD (H, M) or mean (K‐L). *p* values were analyzed by chi‐square (*χ*2) (A), two‐tailed Student's *t*‐test (H, M), and Kaplan‐Meier survival analysis (B, E, F, I, N, O). (*p < 0.05, **p < 0.01, ***p < 0.001).

Previous studies have shown that the presence of circulating tumor cells (CTC) in solid tumors may be a prerequisite for metastasis, and especially mesenchymal circulating tumor cells (MCTC) predicting poor prognosis significantly.^[^ ^9^ ^]^ We analyzed the proportion of MCTC‐positive cells in the peripheral blood of different subtypes of breast cancer patients and found that TNBC patients had a higher proportion of MCTC‐positive cells compared to non‐TNBC patients (**Figure** **1**A). We also found the presence of MCTC in the patients' blood was significantly associated with the poor prognosis of triple negative breast cancer (Figure 1B). Previous studies indicated that CTC require the anoikis resistance mechanism to survive and reach distant metastatic sites.^[^ ^10^ ^]^ We analyzed the anoikis of TNBC and non‐TNBC cells in the suspension culture and found that TNBC cells were much more resistant to detachment‐induced anoikis than non‐TNBC cells (Figure S1A,B, Supporting Information).

To explore the key cellular process that contributes to increased CTC rates and the malignant progression of TNBC, we compared the expression profiles of TNBC, HR^+^, and HER2^+^ subtype tumor tissues in TCGA breast cancer dataset (Figure 1C). The differential gene set enrichment analysis showed that focal adhesion (FA) and regulation of actin cytoskeleton pathways were enriched in TNBC tissues (Figure 1D). Polygenic risk analysis demonstrated that high risk level of the FA pathway significantly correlated with poor prognosis of breast cancer patients (Figure 1E; Figure S1C,D, Supporting Information). Furthermore, among the key proteins in FA pathways, FAK, also known as PTK2, exhibited the most significant impact on the overall survival of TCGA breast cancer patients (Figure 1F).

Considering the crucial role of FAK in CTC survival in circulation and colonization to distant organs,^[^ ^11^ ^]^ we analyzed the expression of FAK in breast cancer cell lines and tissues. We found that FAK protein levels were higher in TNBC cells compared to non‐TNBC cells (Figure S1E, Supporting Information). Immunohistochemistry (IHC) also revealed that FAK expression was higher in TNBC tissues compared to non‐TNBC tumor tissues (Figure 1G,H). Moreover, high FAK protein level was associated with poor overall survival of breast cancer patients (Figure 1I). These data suggest the significant role of FAK protein in the progression and metastasis of TNBC.

### 2.2 FAISL is Highly Expressed in TNBC and Associated with FAK

Previous studies have shown that FAK binds to Src, Paxillin, p130CAS, and α‐actinin to form the focal adhesion complex to promote cell adhesion, migration, and survival.^[^ ^4^, ^12^ ^]^ To investigate whether lncRNAs are involved in the regulation of FAK signaling pathways, we performed RNA immunoprecipitation (RIP) to screen FAK‐associated lncRNAs in TNBC cells. We overexpressed Flag‐FAK in MDA‐MB‐231 cells and precipitated RNA using the Flag antibody. Compared to IgG, 34 lncRNAs were enriched more than twofolds in the Flag‐FAK precipitates (Figure 1J). RIP and RT‐qPCR validation showed that lncRNA VPS9D1‐AS1, which we later named FAISL, was enriched in both MDA‐MB‐231 and MDA‐MB‐468 cells (Figure 1K,L).

Next, we analyzed FAISL expression in breast cancer datasets. Similar with FAK, FAISL expression was higher in TNBC tissues than non‐TNBC and normal breast tissues in the TCGA breast cancer database (Figure 1M). In consistence, FAISL expression was lower in non‐TNBC cell lines than TNBC cell lines in TCGA database and our cultured cells (Figure S1F,G, Supporting Information). Then we performed survival analysis in TCGA database and found that FAISL was not significantly associated with the overall survival and disease‐free survival (DFS) in the breast cancer cohort (Figure S1H,I, Supporting Information). As in the TCGA dataset the tumors with neoadjuvant treatment could not be excluded, we analyzed FAISL expression in the KM‐Plotter RNA‐seq database, where we could select the neoadjuvant treatment‐naïve tumor tissues. Kaplan‐Meier analysis showed that high expression of FAISL predicted poor overall survival in the breast cancer cohort (all subtypes together) (*p* = 1.3e‐05, hazard ratio (HR) = 3.09) and the TNBC cohort (*p* = 0.022, HR = 3.77) (Figure 1N,O). Additionally, the expression of FAISL was associated with the overall survival of head‐neck squamous cell carcinoma (*p* = 0.0088, HR = 1.53), kidney renal clear cell carcinoma (*p* = 1.9e‐12, HR = 2.82), liver hepatocellular carcinoma (*p* = 0.00092, HR = 1.84), lung adenocarcinoma (*p* = 0.015, HR = 1.43), pheochromocytoma and paraganglioma (*p* = 0.021, HR = 728 809 159.77), and bladder carcinoma (*p* = 0.029, HR = 1.39) (Figure S1J–O, Supporting Information) in the KM‐Plotter database.

### 2.3 Manipulation of FAISL Expression Exhibits Phenotypes Reminiscent of FAK in TNBC Cells

FAISL is located on human chromosome 16 with low interspecies conservation. To determine the sequence and expression of FAISL in breast cancer cells, we performed 5′ and 3′ RACE (Figure S2A, Supporting Information) and verified that FAISL is a 1753 nt transcript with 4 exons and poly‐A tail (Figure S2B, Supporting Information), which was consistent with the sequence in UCSC Genome Browser database. FAISL is a non‐coding transcript, as the protein coding potential score is −1.0, which was calculated by the coding potential calculator software PLEK.^[^ ^13^ ^]^ Nuclear and cytoplasm fractionation followed by RT‐qPCR demonstrated that FAISL was mainly in the cytoplasm of breast cancer cells (Figure S2C,D, Supporting Information). Confocal microscopy of fluorescence in situ hybridization (FISH) for FAISL and immunofluorescence (IF) staining for FAK showed the colocalization of FAISL and FAK in the cytoplasm of MDA‐MB‐231 cells (Figure S2E, Supporting Information).

**Figure 2:**
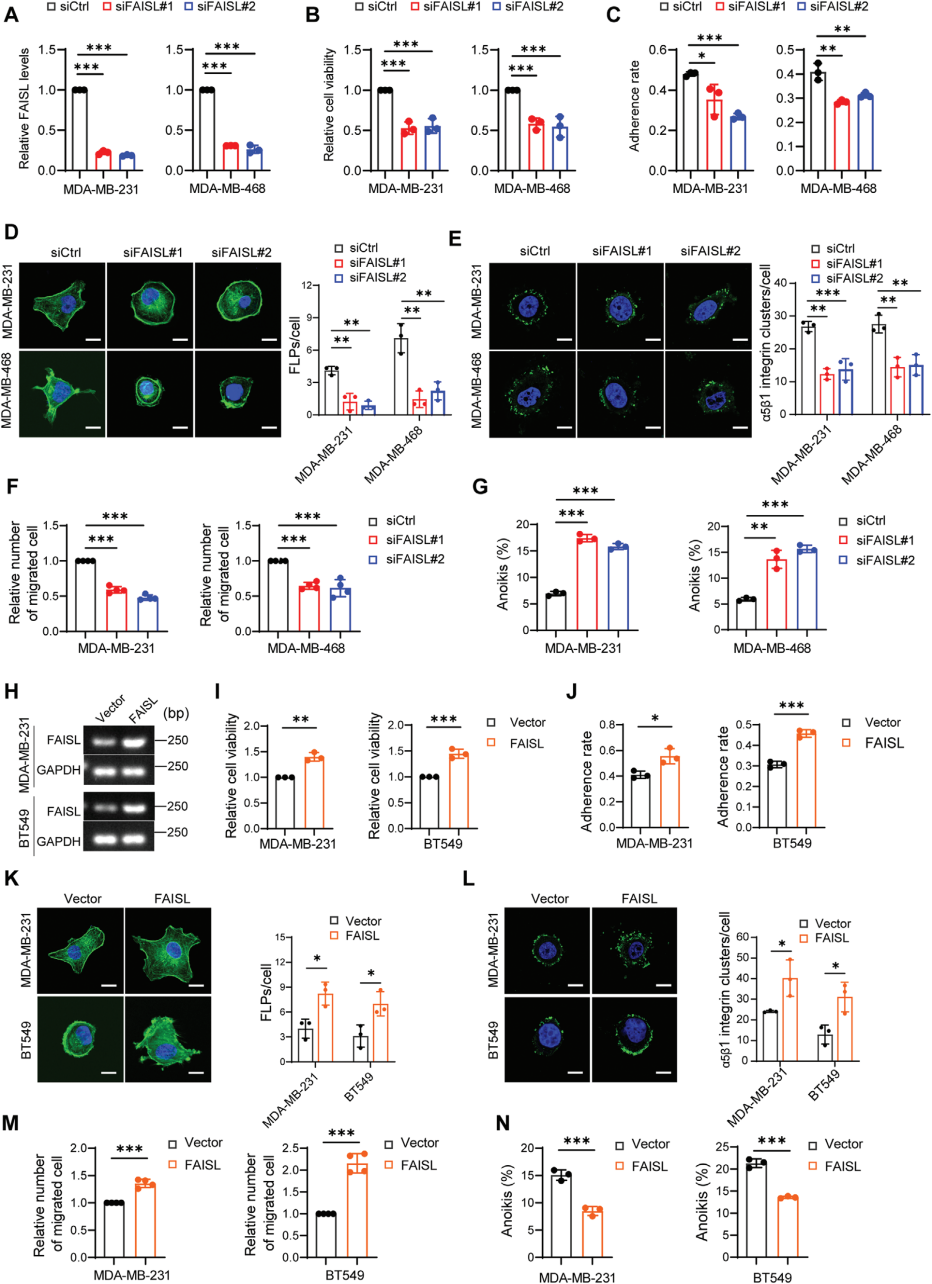
FAISL promotes cell viability, adhesion, migration, and anoikis resistance. A) RT‐qPCR shows FAISL knockdown efficiency by siRNAs in MDA‐MB‐231 and MDA‐MB‐468 cells. B) FAISL knockdown reduces cell viability 48 h post transfection. C) FAISL knockdown reduces cell adhesion in plates coated with fibronectin (FN). D) Fluorescent phalloidin (F‐actin) shows filopodium‐like protrusions (FLPs) in siCtrl and siFAISL cells. Scale bar, 10 µm. E) Representative fluorescence staining and quantification of α5β1 integrin clusters (green) in cells grown on FN‐coated plates for 30 min. Scale bar, 10 µm. F) FAISL knockdown reduces cell migration through the transwell. G) FAISL knockdown increases the detachment‐induced anoikis. Cells were cultured in suspension for 12 h and subjected to apoptosis analysis. H) Exogenous expression of FAISL in MDA‐MB‐231 and BT549 cells. I) FAISL overexpression promotes cell viability 48 h post stable infection. J) FAISL overexpression promotes cell adhesion in plates coated with fibronectin (FN). K) Fluorescent phalloidin (F‐actin) shows filopodium‐like protrusions (FLPs) in cells. Scale bar, 10 µm. L) Representative fluorescence staining and quantification of α5β1 integrin clusters (green) in cells grown on FN‐coated plates for 30 min. Scale bar, 10 µm. M) FAISL overexpression promotes cell migration through the transwell. N) FAISL overexpression decreases the detachment‐induced anoikis. Cells were cultured in suspension for 24 h and subjected to apoptosis analysis. Data are presented as mean ± SD of experimental triplicates (A–E, G, I–L, N) or quadruplicates (F, M). *P* values were assessed with two‐tailed Student's *t*‐test. (*p < 0.05, **p < 0.01, ***p < 0.001).

To elucidate the role of FAISL in TNBC cells, we knocked down FAISL using siRNAs in MDA‐MB‐231 and MDA‐MB‐468 cells (**Figure** **2**A). Silencing FAISL significantly inhibited the proliferation (Figure 2B) and the adhesion of cells to the extracellular matrix (ECM) (Figure 2C). Immunofluorescence staining showed that silencing FAISL inhibited F‐actin skeleton spreading (Figure 2D) and the aggregation of α5β1 integrin (Figure 2E). The transwell assay demonstrated that knocking down FAISL reduced the migration of TNBC cells (Figure 2F; Figure S2F, Supporting Information). In low‐attachment cell culture plates, knocking down FAISL increased the anoikis of both MDA‐MB‐231 and MDA‐MB‐468 cells (Figure 2G; Figure S2G, Supporting Information).

When exogenously expressing FAISL in low FAISL‐expressed BT549 and moderate FAISL‐expressed MDA‐MB‐231 cell lines (Figure 2H), we found FAISL promoted the anchorage‐dependent cell proliferation, cell adhesion to the extracellular matrix, cytoskeleton spreading, integrin clustering and migration (Figure 2I–M; Figure S2H, Supporting Information). Moreover, apoptosis analysis showed that overexpression of FAISL also reduced the anoikis in BT549 and MDA‐MB‐231 cells (Figure 2N; Figure S2I, Supporting Information). Together, these data demonstrated that FAISL knockdown or overexpression induced phenotypes reminiscent of manipulating FAK expression in cell proliferation, adhesion, migration, and anoikis resistance.

### 2.4 FAISL Stabilizes FAK Protein by Interacting with Calpain 2

**Figure 3:**
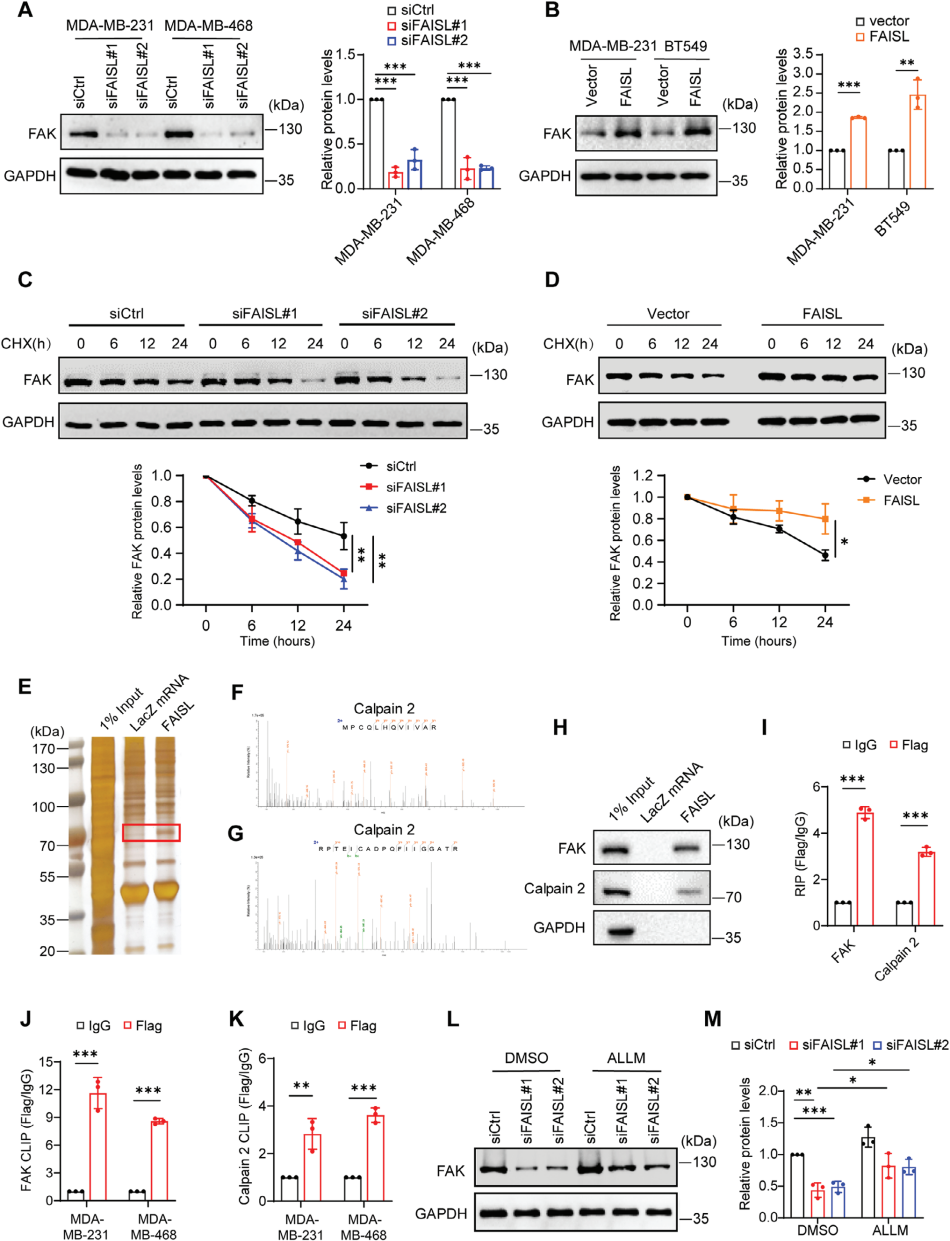
FAISL stabilizes FAK protein and binds to Calpain 2. A–B) Western blot and quantification of FAK protein in TNBC cells after FAISL knockdown (A) or overexpression (B). C–D) Cycloheximide (CHX) chase assay of FAK in MDA‐MB‐231 cells. 100 µg mL^−1^ CHX was added to the culture medium after FAISL knockdown (C) or overexpressing (D). E) RNA pulldown using biotin‐labeled FAISL and the LacZ mRNA (negative control) in MDA‐MB‐231 cells. The differential bands of the silver staining gel in the red frame were sent for mass spectrometry identification. F–G) Mass spectrometry profiles of FAISL‐binding protein Calpain 2. H) RNA pulldown followed by western blot shows that FAISL binds to FAK and Calpain 2 in MDA‐MB‐231 cells. I) RNA immunoprecipitation followed by RT‐qPCR in MDA‐MB‐231 cells using the anti‐Flag antibody. MDA‐MB‐231 cells were transfected with Flag‐FAK or Flag‐Calpain 2. J–K) Cross‐linking immunoprecipitation (CLIP) and RT‐qPCR in MDA‐MB‐231 and MDA‐MB‐468 cells with the anti‐Flag antibody. Cells were transfected with Flag‐FAK (J) or Flag‐Calpain 2 (K). L–M) Western blot (L) and quantification (M) of FAK protein in MDA‐MB‐231 treated with ALLM (10 µg mL^−1^, 12 h). Data are presented as mean ± SD of experimental triplicates (A–D, I–K, M). *p* values were determined using two‐tailed Student's *t*‐test (A–D, I–K, M). (*p < 0.05, **p < 0.01, ***p < 0.001).

When exploring the impact of FAISL binding to FAK, we found that knocking down FAISL dramatically decreased the protein level of FAK in MDA‐MB‐231 and MDA‐MB‐468 cells (**Figure** **3**A). Consistent with the knockdown results, overexpression of FAISL in BT549 and MDA‐MB‐231 cells increased the protein level of FAK (Figure 3B). However, knockdown or overexpression of FAISL did not affect the mRNA levels of FAK or VPS9D1, the protein‐coding sense gene of FAISL (Figure S3A–D, Supporting Information). In TCGA breast cancer database, FAISL shows little correlation with FAK mRNA (*R* = 0.073) (Figure S3E, Supporting Information), indicating that FAISL doesn't regulate FAK at the mRNA level.

Next, we explore whether FAISL affect FAK protein level by regulating its stability. Cycloheximide (CHX) chase assay demonstrated that knocking down FAISL reduced FAK stability in MDA‐MB‐231 cells (Figure 3C). Consistently, overexpression of FAISL in MDA‐MB‐231 increased FAK stability (Figure 3D). These results indicate that FAISL increases FAK protein levels by stabilizing FAK.

Previous studies have shown that FAK protein stability can be regulated by the E3 ligase c‐Cbl mediated ubiquitination, as well as Calpain or Caspase‐mediated cleavage.^[^ ^3^, ^14^ ^]^ To further study the mechanism of how FAISL regulated FAK, we performed RNA pulldown to identify FAISL‐interacting proteins. Compared to the LacZ mRNA negative control, a band between 70 ∼ 100 kDa was enriched in FAISL ‐pulldown complex (Figure 3E). Mass spectrometry (MS) identified multiple proteins in this band and Calpain 2, a member of the Calpain family, ranked top of the list (Figure 3F,G; Figure S3F, Supporting Information).

Calpain 2 is a cysteine protease that has been shown to cleave cytoskeletal and submembranous proteins, such as FAK and Talin, and affect the turnover of focal adhesion.^[^ ^15^ ^]^ Western blots after RNA pulldown showed that FAISL bound not only to FAK but also to Calpain 2 in MDA‐MB‐231 cells (Figure 3H). RIP assay showed that FAISL was retrieved with fivefold and threefold enrichment in the anti‐FAK and anti‐Calpain 2 immunoprecipitates, respectively, compared to IgG in MDA‐MB‐231 cells (Figure 3I). Moreover, in the CLIP assay (cross‐linked condition), the enrichment of FAISL in anti‐Flag FAK and anti‐Flag Calpain 2 immunoprecipitates were even more dramatic than in the RIP assay (Figure 3J,K).

To test whether FAISL affected FAK protein level through Calpain 2‐induced proteolysis, we treated MDA‐MB‐231 cells with Calpain inhibitor N‐acetyl‐Leu‐Leu‐Met (ALLM), which could effectively inhibit Calpain 2 activity.^[^ ^16^ ^]^ We found that ALLM treatment partially reverse the FAK protein level deceased by FAISL knockdown (Figure 3L,M). Together, these results suggested that lncRNA FAISL affected FAK proteolysis by interacting with both Calpain 2 and FAK.

### 2.5 FAISL Binds to FAK C‐Terminus Domain to Inhibit Calpain 2‐Induced FAK Proteolysis

**Figure 4:**
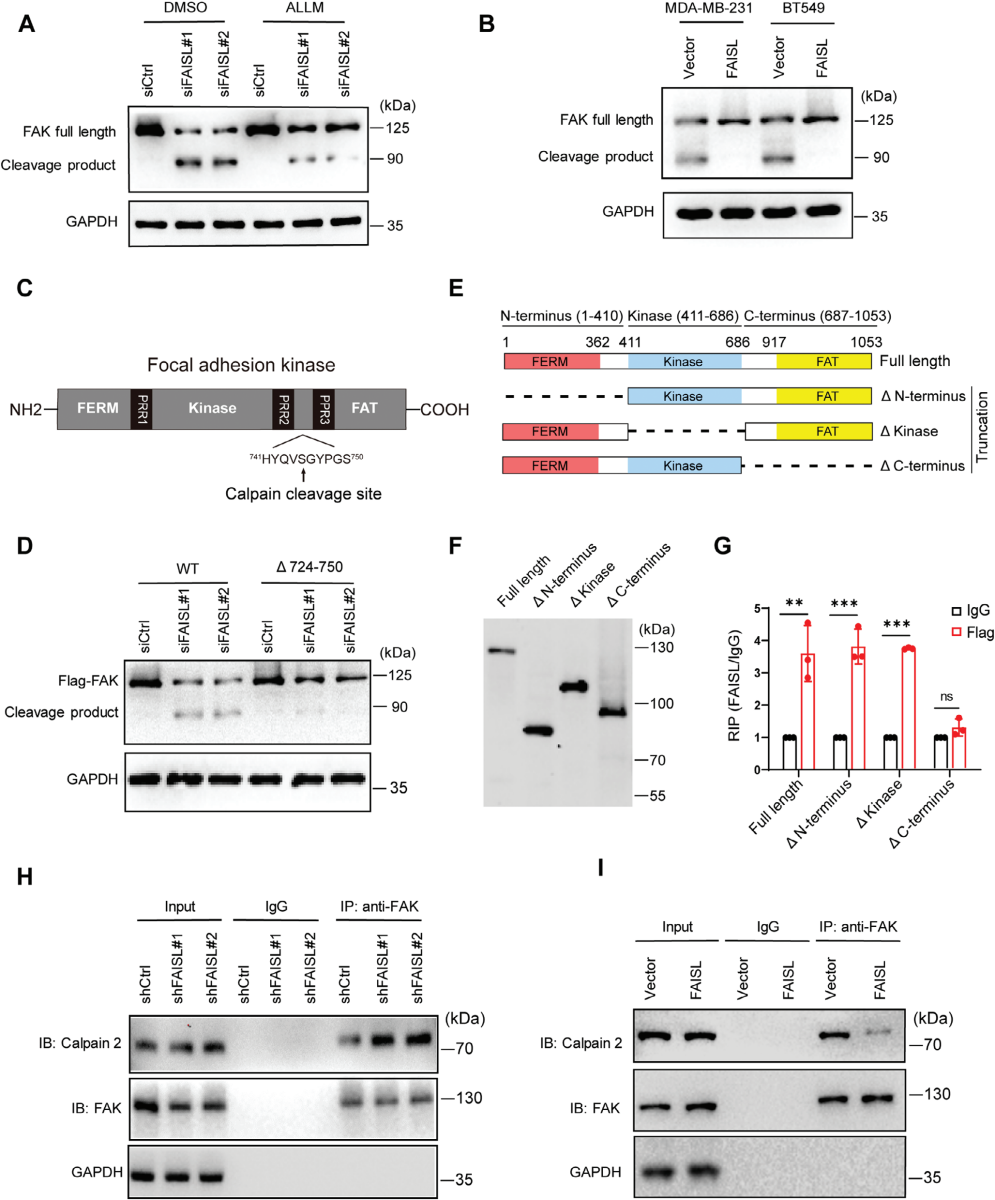
FAISL inhibits Calpain 2‐mediated proteolysis of FAK by masking the cleavage site of FAK. A) Western blot shows the proteolytic cleavage of FAK protein after knocking down FAISL in MDA‐MB‐231 cells treated by ALLM or not. B) Western blot shows the proteolytic cleavage of FAK protein in MDA‐MB‐231 and BT549 cells after overexpressing FAISL. Cells were treated with 1 µM calcium ionophore Ionomycin and 1 mM CaCl₂. C) The schematic diagram of FAK domains denoting the site of Calpain cleavage.^[^ ^14a^ ^]^ D) Western blot shows the cleavage of FAK protein after knocking down FAISL in MDA‐MB‐231 cells. Cells were transfected with wild‐type Flag‐FAK (WT), or Flag‐FAK D724‐750. E) The schematic diagram of full‐length or truncated FAK variants deleting the N‐terminus, Kinase, or C‐terminus. F) Western blot of Flag‐tagged full‐length or truncated FAK variants in MDA‐MB‐231 cells. G) RIP and RT‐qPCR of FAISL using Flag‐FAK (full length or truncation variants) in MDA‐MB‐231 cells. H–I) FAISL affects the interaction of FAK and Calpain 2, as detected by co‐immunoprecipitation and western blot in MDA‐MB‐231 cell lines with stable FAISL knockdown (H) or overexpression (I). Data are presented as mean ± SD of experimental triplicates (G). *p* values were determined using two‐tailed Student's *t*‐test (G). (*p < 0.05, **p < 0.01, ***p < 0.001, ns means not significant).

Calpain 2 can cleave FAK into ≈90 kDa N‐terminal and ≈30 kDa C‐terminal fragments by binding with the C‐terminus domain of FAK.^[^ ^14^, ^17^ ^]^ After knocking down FAISL, a ≈90 kDa band was detected by FAK N‐terminal antibody in MDA‐MB‐231 cells, and this cleavage could be abrogated by ALLM (**Figure** **4**A). Consistent with the knockdown result, ectopic expression of FAISL in MDA‐MB‐231 and BT549 cells decreased the cleavage of FAK induced by ionomycin and CaCl₂ (Figure 4B), which increased the intracellular calcium ion level and the cleavage activity of Calpain 2.^[^ ^18^ ^]^ Moreover, we constructed a Flag‐tagged truncated mutant of FAK by deleting the residues around Calpain 2 cleavage site in the C‐terminus domain (D724‐750) (Figure 4C).^[^ ^17^ ^]^ FAISL knockdown increased the cleavage of wildtype Flag‐FAK, whereas D724‐750 was resistant to the cleavage, which confirmed that FAISL‐involved FAK cleavage was mediated by Calpain 2 (Figure 4D).

To map the domain of FAK binding to FAISL, we generated Flag‐FAK containing full length or truncation mutants deleting the N‐terminus, kinase domain, C‐terminus respectively (Figure 4E), as verified by immunoblots (Figure 4F). RNA IP using Flag antibody and subsequent RT‐qPCR showed that deletion of the C‐terminus domain, but not other domains, specially abrogated the interaction of FAK with FAISL (Figure 4G), suggesting that FAISL might mask the binding site of Calpain 2 on FAK. To further explore whether FAISL affect the interaction of FAK and Calpain 2, we performed Co‐IP using FAK antibody in MDA‐MB‐231 cells. Immunoblots following IP showed that silencing FAISL increased the binding of Calpain 2 to FAK (Figure 4H), while overexpressing FAISL reduced the binding of Calpain 2 to FAK in MDA‐MB‐231 cells (Figure 4I). Together, lncRNA FAISL inhibited Calpain 2‐mediated proteolysis of FAK by masking the cleavage site of Calpain 2 on FAK.

### 2.6 The siRNA Nanodelivery System Targeting FAISL Effectively Suppresses Malignant Phenotypes of TNBC Cells

**Figure 5:**
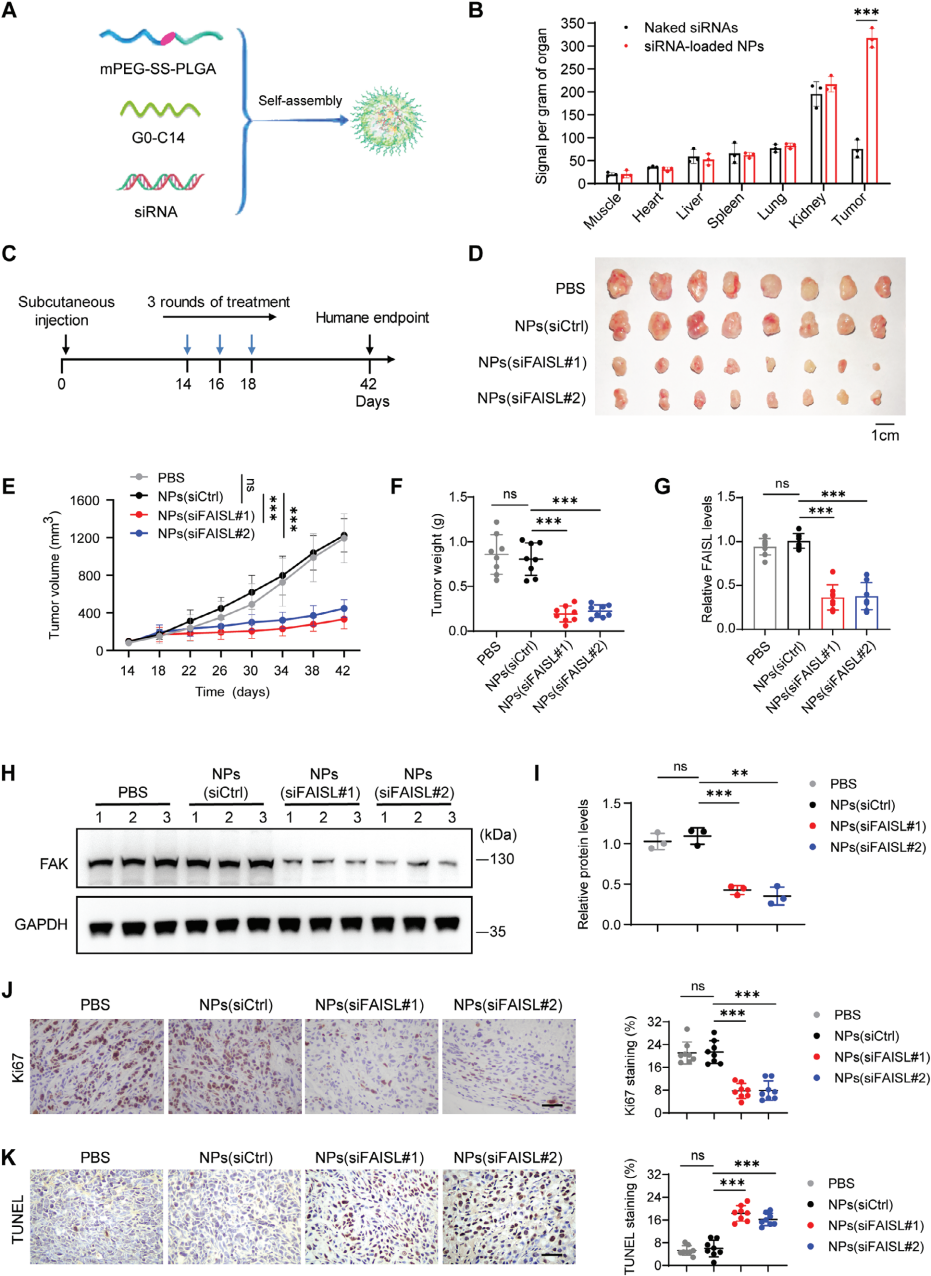
The siRNA nanodelivery system targeting FAISL inhibits the growth of TNBC xenografts in nude mice. A) Schematic diagram of the NPs (siRNA) made with meo‐PEG‐S‐S‐PLGA and cationic lipid G0‐C14. B) Biodistribution of siRNA‐loaded NPs and naked siRNA in nude mice. C) The treatment schedule of MDA‐MB‐231 xenografts in nude mice. 14 days after subcutaneous tumor inoculation, mice were treated with PBS, NPs (siCtrl), NPs (siFAISL#1), and NPs (siFAISL#2) at a 1 nmol siRNA dose treatment per mouse. D) Image of collected tumors from the MDA‐MB‐231 tumor‐bearing mice treated with NPs (siRNA) in (C). E) Tumor growth curve of the MDA‐MB‐231 xenografts in nude mice. F) Tumor weights of the MDA‐MB‐231 xenografts in (D). G) FAISL expression determined by RT‐qPCR in the tumor tissues collected in (D). H) FAK expression determined by western blot in the tumor tissues collected in (D). I) The FAK protein levels of tumor tissues were quantified from (H). J–K) Ki67 IHC (J) and TUNEL assay (K) in the MDA‐MB‐231 xenografts. Scale bars, 20 µm. For B, H, I, *n* = 3 mice per group. For C–G, J–K, *n* = 8 mice per group. Each dot represents one mouse (F–G, I–K). For B, E–G, I–K, mean ± SD are shown, and *p* values were determined using two‐tailed Student's *t*‐test (B, E–G, I–K). (*p < 0.05, **p < 0.01, ***p < 0.001, ns means not significant).

As FAISL knockdown could inhibit malignant phenotypes of TNBC cells in vitro, we took advantage of the siRNA nanodelivery system to more effectively target FAISL. We encapsulated the control and FAISL siRNAs into reduction‐responsive nanoparticles (NPs) (**Figure** **5**A; Figure S4A,B, Supporting Information).^[^ ^19^ ^]^ The particle size of the siRNA‐encapsulated nanoparticles was ≈100 nm (Figure S4C, Supporting Information), and the zeta potential was detected by dynamic light scattering instrument (Figure S4D, Supporting Information). Compared with PBS, the release rate of NPs in GSH solution was faster (Figure S4E,F, Supporting Information), as the sulfhydryl group in the GSH molecule can redox the surface of the nanoparticle, resulting in the reduction of the stability of the nanoparticle, thereby promoting its dissociation and release of siRNAs.

We first tested the knockdown efficiency of siRNA‐encapsulated nanoparticles in MDA‐MB‐231 cells, and found NPs (siRNA) at concentrations of 30 nM and 50 nM could knock down FAISL to less than 40% and 30%, respectively (Figure S4G, Supporting Information), which was comparable to that of traditional liposome transfection of siRNAs (Figure S4H, Supporting Information). Then we used siRNA‐encapsulated nanoparticles at the concentration of 50 nM to perform in vitro experiments. Consistent with previous knockdown results, NPs (siFAISL) significantly inhibited cell proliferation (Figure S4I, Supporting Information), adhesion (Figure S4J, Supporting Information), and anoikis resistance of MDA‐MB‐231 cells (Figure S4K, Supporting Information).

### 2.7 The siRNA Nanodelivery System Targeting FAISL Inhibits the In Vivo Growth of TNBC Cells

To further apply nanoparticles to in vivo treatment, we evaluate the blood circulation curve and organ distribution of nanoparticles in nude mice. The NPs (siRNA) (1 nmol siRNA dosage per mouse) displayed a longer blood circulation time due to the outer layer protection, with a half‐life of ≈1.5 h. In contrast, naked siRNA was rapidly cleared from the blood, with a blood circulation half‐life of less than 10 min (Figure S5A, Supporting Information). Furthermore, we collected tumors and major organs (muscle, heart, liver, spleen, lung, and kidney) from nude mice 24 h after injection. We found that both naked siRNA and NPs (siRNA) accumulated in the kidney and liver, but NPs (siRNA) accumulated much more in the tumors compared to naked siRNA (Figure 5B; Figure S5B,C, Supporting Information).

Next, we subcutaneously implanted MDA‐MB‐231 cells in nude mice. 14 days after implantation, mice were intravenously injected with siRNA‐loaded nanoparticles at a dosage of 1 nmol siRNA per mouse every two days for three times of treatments (Figure 5C). The tumor growth curve showed that nanoparticles loaded with siFAISL dramatically inhibited tumor growth, as also confirmed by the tumor weights at the humane endpoint, compared to those treated with PBS or NPs (siCtrl) (Figure 5D–F). Moreover, no significant impact on the organs (heart, liver, spleen, lungs, and kidneys) and body weights of the mice were detected with the treatments of siRNA nanodrug targeting FAISL (Figure S5D–F, Supporting Information).

Furthermore, we collected the xenografts to extract RNA and protein. RT‐qPCR showed that after three consecutive nanoparticle treatments, NPs (siFAISL#1) and NPs (siFAISL#2) could reduce FAISL expression by ≈50% (Figure 5G). Western blot also showed that NPs (siFAISL#1) and NPs (siFAISL#2) significantly downregulated FAK protein levels (Figure 5H,I). Ki67 immunohistochemistry and TUNEL staining demonstrated that the siRNA‐loaded nanoparticles targeting FAISL effectively decreased the number of Ki67‐positive cells (Figure 5J) and increased the proportion of apoptotic cells in the xenografts (Figure 5K).

### 2.8 The siRNA Nanodelivery System Targeting FAISL Inhibits Distant Metastasis of TNBC Cells

**Figure 6:**
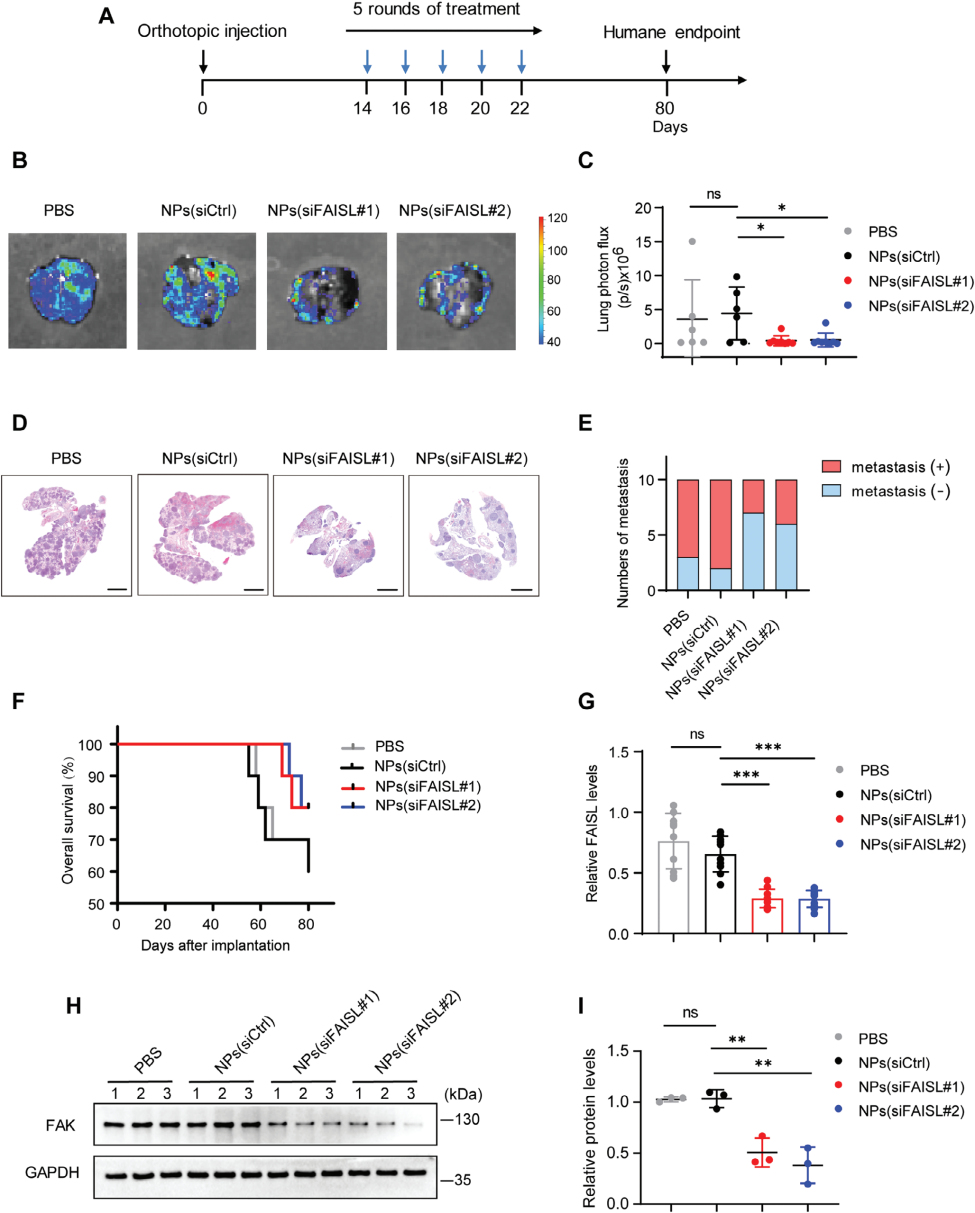
The siRNA nanodelivery system targeting FAISL inhibits the breast cancer metastasis. A) The treatment schedule of xenograft inoculation and NPs (siRNA) treatments. Luciferase‐MDA‐MB‐231 cells were orthotopically injected into NOD/SCID mice. When the tumors were palpable, mice were treated with PBS, NPs (siCtrl), NPs (siFAISL#1), and NPs (siFAISL#2) at a 1 nmol siRNA dose treatment per mouse (*n* = 10). B–C) Representative bioluminescence images (B) and quantification (C) of lung metastasis in NOD/SCID mice with the indicated treatments. D) Representative HE staining of metastatic nodules in the lungs of NOD/SCID mice at the endpoint, *n* = 10 mice per group. Scale bars, 2 mm. E) Quantification of mice with lung metastasis. F) Kaplan‐Meier survival analysis of NOD/SCID mice orthotopically implanted with MDA‐MB‐231 cells (*n* = 10 per group). G) RT‐qPCR analysis of FAISL in the orthotopic xenografts. H–I) Western blot (H) and quantification (I) of FAK in the orthotopic xenografts. Each dot represents one mouse (C, G, I). For C, G, I, mean ± SD are shown, and *p* values were determined using two‐tailed Student's *t*‐test. (*p < 0.05, **p < 0.01, ***p < 0.001, ns means not significant).

To explore the effect of targeting FAISL with siRNA‐loaded nanoparticles on TNBC metastasis, we established a spontaneous lung metastasis model by orthotopically implanting invasive MDA‐MB‐231 cells in NOD/SCID mice. 14 days after implantation, the mice were treated every 2 days with a dose of 1 nmol siRNA per mouse via tail vein injection for a total of 5 consecutive treatments (**Figure** **6**A). At the humane endpoint, lung tissues and orthotopic tumor tissues were collected from the mice. Bioluminescence imaging and HE staining of lung sections showed that compared to the mice treated with PBS or siCtrl, the mice injected with NPs (siFAISL) had a significantly lower incidence of lung metastasis (Figure 6B–E). Kaplan‐Meier survival curve analysis demonstrated that NPs (siFAISL) treatment prolonged the overall survival of the mice compared to the PBS and NPs (siCtrl) treatments (Figure 6F). Moreover, RT‐qPCR of the orthotopic tumor tissues showed that after five consecutive nanoparticle treatments, NPs (siFAISL#1) and NPs (siFAISL#2) inhibited over 50% of FAISL expression compared to the PBS and NPs (siCtrl) groups (Figure 6G). Western blot analysis demonstrated ≈50% reduction of FAK protein with the knockdown of FAISL (Figure 6H,I). These results indicated that nanoparticles targeting FAISL efficiently suppressed FAISL expression and lung metastasis of TNBC cells in vivo.

### 2.9 High Level of FAISL Predicts Poor Prognosis in TNBC Patients

**Figure 7:**
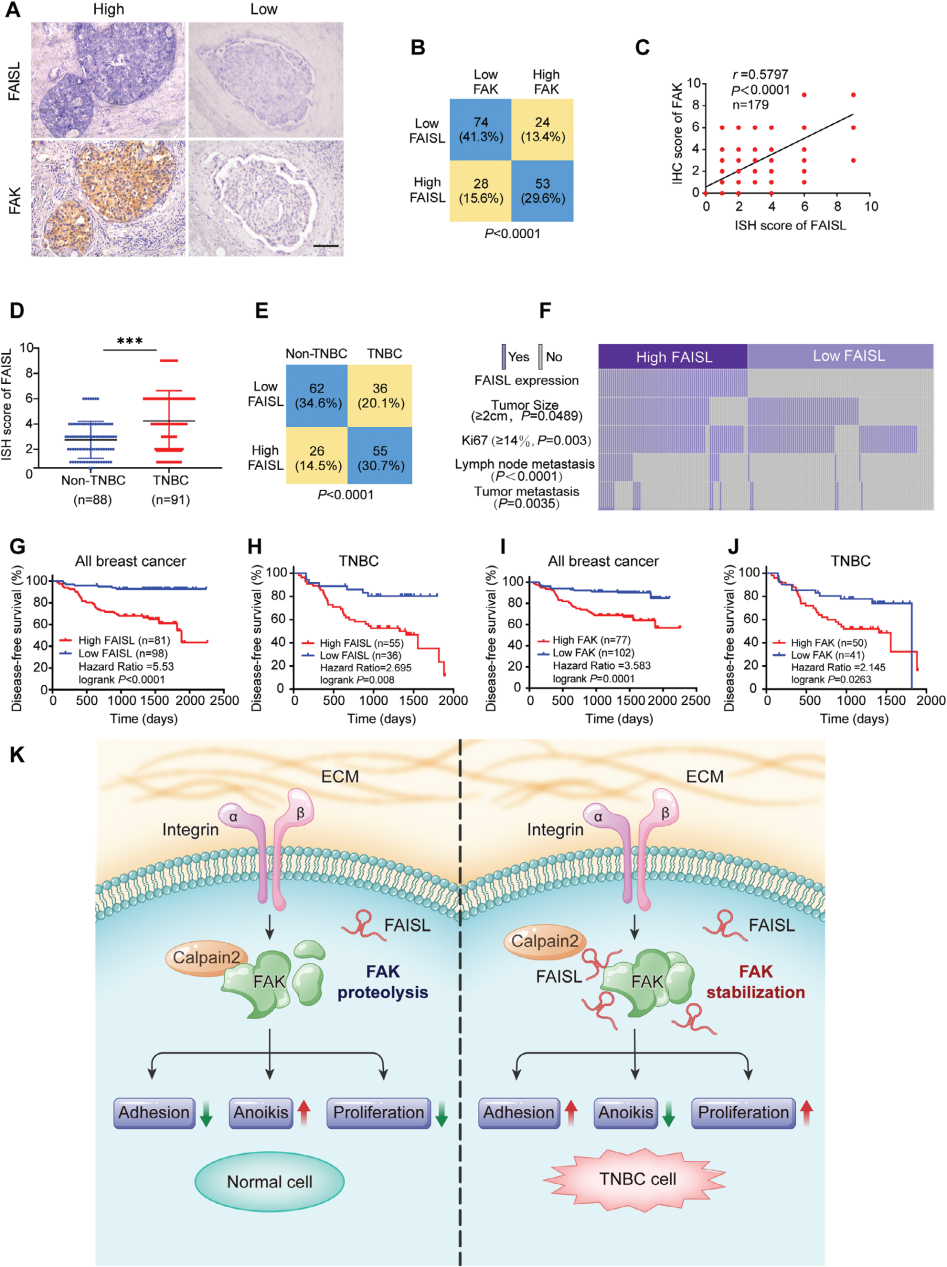
FAISL expression is associated with FAK protein level and poor prognosis of breast cancer patients. A) Representative images of FAISL ISH and FAK IHC of breast cancer tissues. Scale bars, 20 µm. B) The distribution of FAK and FAISL expression, as evaluated by the staining index. C) Spearman correlation analysis of FAISL expression with FAK expression in breast cancer specimens. D) FAISL expression in non‐TNBC and TNBC tissues. E) The distribution of FAISL in the specimen of non‐TNBC (*n* = 88) or TNBC patients (*n* = 91). F) The association of FAISL expression with clinicopathological features of breast tumors. G–H) Kaplan‐Meier analysis for disease‐free survival in all breast cancer (G), and TNBC patients (H) with different FAISL expression in tumor tissues. I–J) Kaplan‐Meier analysis for disease‐free survival in all breast cancer (I), and TNBC patients (J) with different FAK expression in tumor tissues. K) Graphic working model of lncRNA FAISL in promoting TNBC progression and metastasis through enhancing FAK stabilization. *p* values were determined by two‐tailed Chi‐square (*χ*2) test (B, E–F), Spearman analysis (C) two‐tailed Student's *t*‐test (D), or log‐rank test (G–J). (*p < 0.05, **p < 0.01, ***p < 0.001).

To determine the clinical relevance of FAISL and FAK in breast cancer, we detected FAISL and FAK expression in the primary breast cancer tissues. Consistent with the FISH results in MDA‐MB‐231 cells, the ISH assays showed that FAISL mainly located in the cytoplasm of tumor cells, colocalized with FAK protein in the breast cancer tissues (**Figure** **7**A). Tumors in the FAISL‐high group expressed higher levels of FAK than those in the FAISL‐low group (Figure 7B). Spearman correlation analysis showed that FAISL expression was positively correlated with the protein level of FAK (*r* = 0.5797, *p* < 0.0001) (Figure 7C). Besides, TNBC tissues expressed relative higher levels of FAISL than non‐TNBC tissues (Figure 7D), The proportion of tumors with high expression of FAISL was higher in the TNBC group than those in the non‐TNBC group (Figure 7E), indicating that FAISL was a relative specific lncRNA that highly expressed in TNBC tissues.

In the 179 tumor samples of breast cancer patients with follow‐up information, FAISL expression was positively associated with both tumor size (*p* = 0.0489), Ki67 index (*p* = 0.0029), lymph node (*p* < 0.0001) and distant metastasis (*p* = 0.0035) (Figure 7F; Table S4, Supporting Information). Kaplan–Meier survival analysis indicated that high FAISL level was associated with disease‐free survival in all breast cancer (HR = 5.53, *p* < 0.0001) (Figure 7G) and TNBC patients (HR = 2.695, *p* < 0.008) (Figure 7H). Moreover, high FAK protein level was also associated with disease‐free survival (DFS) in all breast cancer (HR = 3.583, *p* = 0.0001) (Figure 7I) and TNBC patients (HR = 2.145, *p* = 0.0263) (Figure 7J). Collectively, the expression of FAISL and FAK was highly linked with each other, both of which were clinically relevant to the poor outcome of TNBC patients.

## 3 Discussion

Previous studies have shown that high expression of FAK is positively correlated with shorter overall survival and progression‐free survival in patients with metastatic tumors.^[^ ^2^ ^]^ Our study found that focal adhesion pathway molecules, especially FAK, are most significantly associated with the poor outcome of TNBC patients. More importantly, we identify a FAK‐interacting lncRNA FAISL which also promotes the development and metastasis of TNBC. FAISL protects FAK from Calpain 2‐induced proteolysis by binding to FAK C‐terminus domain, thereby promoting the proliferation, adhesion, migration and anoikis resistance of TNBC cells (Figure 7K). By encapsulating siRNA nanoparticles targeting FAISL, silencing FAISL reduces the expression of FAK protein and inhibits the tumor growth and metastasis of TNBC cells in vivo. Importantly, overexpression of FAISL in TNBC tissues is associated with high level of FAK protein and poor prognosis of patients.

Previous studies have shown that in colorectal cancer cells, FAISL, which named MYU, is a downstream regulatory factor of c‐myc. FAISL binds to RNA‐binding protein hnRNP‐K to stabilize the expression of CDK6, thereby promoting the G1‐S transition of the cell cycle and playing a key role in the proliferation and tumorigenicity of colon cancer cells.^[^ ^20^ ^]^ Besides, the expression of VPS9D1‐AS1 (gene symbol name of FAISL) in colorectal cancer cells can also reduce CD8^+^ T cell infiltration by enhancing the expression of TGF‐β and ISG.^[^ ^21^ ^]^ In recent years, VPS9D1‐AS1 has been found to be widely expressed in tumor tissues and cells, including esophageal squamous cell carcinoma, colon adenocarcinoma, lung adenocarcinoma, hepatocellular carcinoma, endometrial cancer, prostate cancer, ovarian cancer cells, and exerts different functions through interactions with different proteins/miRNAs.^[^ ^22^ ^]^ Here, we also found that FAISL promotes cell proliferation in breast cancer cells. However, in this study, we uncover a novel mechanism that lncRNA FAISL, which is highly expressed in TNBC, can bind to FAK and inhibit Calpain 2 to proteolyse FAK, thereby promoting cell adhesion, integrin clustering, migration and resistance to anoikis in TNBC cells.

To our knowledge, this is the first study showing that FAK can interact with and be regulated by a lncRNA. By both RIP (native condition) and CLIP (cross‐linked condition), we demonstrated that FAISL was enriched in FAK immunoprecipitated RNAs. When mapping the domain of FAK, we found FAISL interacts with the C‐terminus domain, whereby masks the binding site of Calpain 2. Further experiments showed that FAISL prevents the binding of Calpain 2 to FAK and reduced the FAK cleavage. Proteolysis of FAK by Calpain 2 is an important and specific mechanism for regulating the turnover of focal adhesions. Our findings provide novel mechanistic insight into the accurate control of FAK level in this process and in the progression of TNBC.

Currently, FAK kinase inhibitors are being vigorously developed for cancer treatment, and some of them have reached clinical trials targeting various malignant tumors.^[^ ^23^ ^]^ However, kinase‐independent functions of FAK can't be suppressed by the kinase inhibitors.^[^ ^24^ ^]^ Our study elucidates that FAISL specifically blocks FAK proteolysis and stabilizes FAK protein, indicating that FAISL may serve as a novel therapeutic target in the FAK pathway, which has the advantage of inhibiting both kinase‐dependent enzymatic function and kinase‐independent scaffolding function of FAK.

As increasing evidence has demonstrated that lncRNAs are specific targets for cancer therapies, siRNA‐based technologies are promising in targeting the cytoplasmic lncRNAs.^[^ ^25^ ^]^ In our work, we have developed thiol‐disulfide bond‐reducible nanoparticles for in vivo siRNA delivery and cancer therapy. Based on the fact that the concentration of glutathione (GSH) in tumor cells is approximately 100–1000 times higher than that in the extracellular fluid, GSH can effectively degrade disulfide bonds and release encapsulated siRNA, thereby achieving efficient intracellular siRNA delivery and excellent anticancer effects.^[^ ^26^ ^]^ Based on the oncogenic role and high level of FAISL expression in TNBC, we use a reducible nanoparticle system for in vivo delivery of siFAISL and evaluate its clinical translational potential in inhibiting the progression and metastasis of TNBC. In subcutaneous xenograft models, this targeted FAISL‐reducible RNAi nanoparticle platform exhibits prolonged circulation time and high tumor accumulation. In subcutaneous xenografts or orthotopic metastatic models, GSH‐triggered intracellular release of siFAISL efficiently silenced FAISL expression and blocked FAK signaling, providing good therapeutic effects in the animal model of TNBC.

Recently, small molecular compounds have been applied to target lncRNA.^[^ ^27^ ^]^ Besides structure‐based design and high throughput screening, online databases, such as ncRNADrug, NoncoRNA and D‐lnc,^[^ ^28^ ^]^ have provided analytical platforms of lncRNA‐drug interactions and experimental data of drug treatments on lncRNA expressions, which may facilitate the development of small molecular inhibitors that effectively targets FAISL.

In conclusion, our findings reveal a previously unrecognized role of lncRNA FAISL in promoting TNBC progression by blocking Calpain 2‐mediated proteolysis and stabilizing FAK protein. Our study also highlights the potential of targeting FAISL with nanoparticle‐based siRNA for the treatment of TNBC. Further research is needed to explore the clinical application of FAISL‐targeted therapies in TNBC and other types of cancer.

## 4 Experimental Section

### CTC Analysis

Circulating tumor cell (CTC) analysis was performed using FISH assay and CellSearch system. The CTC status mentioned in this study refers to the mesenchymal circulating tumor cells. Based on the number of MCTC, MCTC = 0 per 7.5 mL of peripheral blood as the negative group and MCTC > 0 as the positive group were defined. All CTC data were collected from breast cancer patients approved by Institute Research Ethics Committee of Sun Yat‐sen Memorial Hospital, Sun Yat‐sen University (SSKY‐2024‐669‐01).

### Polygenic Risk Model Construction and Prognosis Analysis

The gene expression and patients’ information were downloaded from TCGA breast cancer dataset. Focal adhesion pathway related genes were extracted from the Molecular Signatures Database (MSigDB). Risk scores were calculated according to the coefficient and expression value of each focal adhesion pathway gene which is significant in multivariate Cox regression. Risk‐groups were deemed as two groups (high‐risk group and low‐risk group) according to the median value of risk scores. Kaplan‐Meier curve and log‐rank test were used to assess the predictive value of risk group. The risk scores of TCGA samples were listed in the Supporting Information.

### Cell Culture and Treatments

Cell lines were purchased from American Type Culture Collection Manassas (ATCC), grown in Dulbecco's modified Eagle medium (DMEM, Gibco) / RPMI 1640 medium (Gibco) supplemented with 10% fetal bovine serum (FBS, Vigonob). All cells were cultured in the incubator at 37 °C with 5% CO2 according to standard protocols.

ALLM (Calpain Inhibitor II, A2603) and Ionomycin (B6947‐10) were purchased from APExBIO and dissolved in dimethyl sulfoxide (DMSO, Sigma‐Aldrich). Matrigel (356234, Corning Inc., USA) was purchased from Corning. Fibronectin (F2006) was purchased from Sigma Aldrich.

### siRNAs and Constructs

Custom‐designed FAISL siRNAs (siFAISL#1 and siFAISL#2, RIBOBIO, China) and negative control siRNA (siCtrl, RIBOBIO, China) were transfected using siRNA‐Mate reagent (G04002, Jima, Suzhou, China) 8–12 hours after seeding. The siRNAs for FAISL were listed in Table S1 (Supporting Information).

For overexpression, FAISL was cloned into pCDH‐puro plasmids, then co‐transfected with D8.9 and VSVG plasmids into the HEK293T cells to generate lentivirus. Cells were transduced with indicated lentivirus and polybrene (4 µg mL^−1^) to overexpress FAISL according to the manufacturer's manuals. Empty pCDH‐puro plasmid was used as the negative control. After 48 hours, the transduced cells were selected with puromycin (1 µg mL^−1^).

Full length or deletion mutant of FAK were cloned into pcDNA3.1 vector with Flag tag. The deletion mutant of FAK were DN‐terminus (deleting aa1‐410), DKinase (deleting aa411‐686), DC‐terminus (deleting aa687‐1053), and D724‐750 (deleting aa724‐750).

### RNA Extraction and RT‐qPCR

RNA was extracted by TRIzol (GLPBIO, GK20008), homogenized in chloroform, and then purified by isopropanol and ethanol. Total RNA (500 ng) was transcribed into first‐strand cDNA using PrimeScript RT Master Mix kit (TAKARA, RR036A). Quantitative real‐time PCR was performed using UNICONTM qPCR SYBR Green Master Mix (11198ES08, Yisheng, Shanghai, China) on LightCycler 480 system (Roche) according to operator's instructions. The CT method was used to calculate the relative levels of target genes, and the data were normalized according to GAPDH or β‐actin levels. Primer sequences were listed in Table S2 (Supporting Information).

### RNA Immunoprecipitation (RIP) and Cross‐Linking RNA Immunoprecipitation (CLIP)

For RNA immunoprecipitation (RIP), cells were washed twice in cold PBS and resuspended in 600 µL lysis buffer including Recombinant RNasin Ribonuclease Inhibitor (Promega, N2515) and Halt Protease and Phosphatase Inhibitor Cocktail (ThermoFisher, 78 446), incubated at 4 °C with slow vortex for 30 min, and then centrifugated at 12 000 × g for 15 min. The lysates were incubated with primary antibody‐coupled magnetic beads for 4 h at 4 °C. Then the RNA in immunoprecipitated complex was extracted, and reverse transcribed into cDNA for qPCR or RNA sequencing. The RIP sequencing data were deposited in GEO database with the accession number GSE268194.

For cross‐linking RNA immunoprecipitation (CLIP), cells were treated with 0.3% paraformaldehyde to cross‐linking protein‐RNA complexes in vitro for 10 min, and then terminated with 0.125 M glycine for 5 min at room temperature. Then the immunoprecipitation assay was performed as described above.

### Rapid Amplification of cDNA Ends (RACE)

SMARTer RACE 5′/3′ Kit (TAKARA, 634 858) was used to determine the full length of FAISL and the possible transcripts in breast cancer cells. RNA was extracted from MDA‐MB‐468 cells and converted into cDNA using the reverse transcriptase. The 5′ and 3′ amplification was performed according to manufacturer's instructions. The amplification products were analyzed by agarose gel electrophoresis and the expected bands were purified using Gel Extraction Kit. The purified products were cloned into the linearized pRACE vector and identified by sequencing the gene‐specific primers designed for the PCR of RACE assay were listed in Table S3 (Supporting Information).

### Nucleus and Cytoplasm Fractionation

To determine the localization of lncRNA FAISL, the nucleus and cytoplasm fractionation of cultured cells were isolated using the PARIS kit (Thermo Scientific, AM1921). According to the manufacturer's manual, 1 × 10^6^ cells were resuspended in 300 µL ice‐cold Cell Fractionation Buffer, and incubated on ice for 5 min, centrifuged for 3 min at 4 °C of 500 × g. The cytoplasmic fraction was then aspirated away from the nuclear pellet, added TRIzol LS Reagent (GLPBIO, GK20009) to extract RNA and analysed isolated nuclear and cytoplasmic fractions by RT‐qPCR respectively.

### Fluorescence In Situ Hybridization (FISH)

Probes targeting FAISL were custom designed and purchased from Biosearch Technologies. For FISH analysis, cells were cultured in 24‐well plate and fixed by 4% formaldehyde, permeabilized in fixing solution containing Recombinant RNasin Ribonuclease Inhibitor (Promega, N2515). RNA was hybridized in hybridization buffer containing the fluorescent probes of FAISL for 10 hours at 37 °C in a moist plate according to the manufacturer's protocol. Then cells were stained with DAPI, and observed by SP8 lightning Confocal Microscope (Leica) and Leica 4.0 data acquisition software.

### Patient Tumor Samples and In Situ Hybridization (ISH)

All tumor samples were collected from breast cancer patients with informed consent, and approved by Institute Research Ethics Committee of Sun Yat‐sen Memorial Hospital, Sun Yat‐sen University (Approval Number SYSKY‐2024‐459‐01).

The expression of FAISL in paraffin‐embedded breast cancer tissue sections was examined with ISH. The probe used for ISH was custom designed and purchased from Exiqon. The tissue samples were dewaxed at 70 °C for 1 h, then treated with xylene and gradient alcohol, blocked in PBST with 10% sheep serum (Boster, China), and hybridized in hybridization buffer (Boster, China) with 25 nM FAISL probe at 50 °C for 16–18 h. The sections were then incubated with Biotin‐conjugated anti‐digoxigenin antibody (diluted 1:1000 in blocking reagent) at 4 °C, and developed using 4‐nitroblue tetrazolium (NBT) and 5‐brom‐4‐chloro‐30‐Indolyl‐phosphate (BCIP) substrate (Beyotime, C3206) at 37 °C. The RNA ISH probe was listed in Table S1 (Supporting Information). The staining index (SI) of FAISL was evaluated as the multiplication of the staining intensity and proportion scores of positively stained cells by counting at least 10 random fields (objective × 40). In detail, the staining intensity was graded into four scales (0, no FAISL staining; 1, weak FAISL staining; 2, moderate FAISL staining; 3, strong FAISL staining). The proportion of FAISL positively stained tumor cells was graded into three levels (0, no FAISL‐positive cells; 1, < 25% FAISL‐positive cells; 2, 25%–50% FAISL‐positive cells; 3, > 50% FAISL‐positive cells). The expression of FAISL was calculated from 0 to 12 according to the SI, with an optimal cutoff value of ≤ 3 (low) versus > 3 (high).

### Immunofluorescence Staining and Immunohistochemistry

For Immunofluorescence staining of integrin, 1 × 10^3^ breast cancer cells were seeded on Fibronectin (FN) coated coverslips inside 24‐well plates overnight prior to the experiment. After washing with cold PBS, cells were fixed with 4% paraformaldehyde and permeabilized with 0.5% Triton X‐100, blocked by PBST containing 10% normal goat serum. The specific primary antibody against α5β1 (Merck millipore, MAB1969) was used to stain integrin clustering, which were followed by secondary antibody conjugated with Alexa Fluor 488 (A‐11008, Invitrogen). Besides, cells were stained with DyLightTM 488 Phalloidin (Cell Signaling Technologies, 12935S) (diluted 1:200) to visualize the cytoskeleton, counterstained with DAPI, and imaged by SP8 lightning Confocal Microscope (Leica) and Leica 4.0 data acquisition software.

For immunohistochemistry of paraffin‐embedded breast cancer tissues, all slides were dewaxed and boiled for antigen retrieval in citric acid buffer. Slides were soaked in 3% H2O2 for 10 min to eliminate endogenous peroxidase and blocked with 10% normal goat serum at room temperature for 30 min. Primary antibodies against FAK (CST, 71433S) (1:500) and Ki67 (CST, 9449T) (1:1000) were incubated overnight at 4 °C, and secondary antibodies for 60 min at 37 °C. Slides were stained with diaminobenzidine (DAB) reagent for 2–3 min and counterstained with hematoxylin. The staining index (SI) was evaluated the same as the ISH results. The expression of FAK was calculated from 0 to 12 according to the SI, with an optimal cutoff value of ≤ 3 (low) versus > 3 (high).

### TUNEL Assay

The assay was performed following the manufacturer's protocol of the TUNEL kit (E‐CK‐A331, Elabscience, Wuhan, China). Paraffin‐embedded tissues slides were treated with 1× protease K for 30 min post‐deparaffinization and rehydration, and then blocked at room temperature for 10 min. Subsequently, TdT enzyme was added and incubated for 60 min at 37 °C. Following TdT incubation, slides were treated with streptavidin‐HRP for 30 min. Finally, slides were stained with diaminobenzidine (DAB) reagent for 2–3 min and counterstained with hematoxylin, and examined using an optical microscope (Leica).

### Cell Viability Assay and Adhesion Assay

Cells were seeded in 96‐well plates. After 0, 1, 2, and 3 days of seeding, cells were harvested and cell viability was measured by the CellTiter‐Glo Luminescent Cell Viability Assay kit (Promega) according to the manufacturer's protocol. 96 well‐plates were coated with 50 µL Fibronectin (FN, Sigma‐Aldrich) in PBS (1x) per well overnight at 4 °C for the adhesion assay. 100 µL cell suspension at 0.1‐2.0 × 10^6^ cells mL^−1^ were seeded into FN coated plates the next day, and incubated in serum‐free medium at 37 °C for 30–60 min. Then the non‐adherent cells were washed away gently with PBS for 3 times. The percentage of adherent cells was measured by CellTiter‐Glo Luminescent Cell Viability Assay kit (Promega) according to manufacturer's protocol.

### Transwell Assay

Cell migration assay was performed with Boyden chamber (Corning, 3422) with 8.0‐µm pore polycarbonate membrane. 600 µL medium containing 20% fetal bovine serum (Vigonob) was added to the lower chamber of 24‐well plate, and the companion chamber was placed over the wells. 100 µL cell suspension at 10^6^ cells mL^−1^ was added to the upper chambers, and the plate was incubated for 12–24 h at 37 °C and 5% CO2. The cells of the membrane upper surface were scraped, and the migrated cells of the lower membrane surface were stained with crystal violet, counted in microscope. The quantification of transwell assay was conducted by Image‐J (National Institutes of Health).

### Anoikis Assay

Cells were added to the low attachment plate (Costar 6 Well Low Attachment Multiple Well Plates, Corning, 3471) after indicated treatment. Cells were cultured for 12–24 h, harvested and washed with cold PBS, and stained with Annexin V‐FITC/PI for 15 min using Annexin V‐FITC/PI apoptosis assay kit (YuanYe, 70‐AP101, China), and detected by flow cytometry (CytoFLEX S, Beckman Coulter, CA).

### Western Blot

Cells were lysed in RIPA buffer (Beyotime, Shanghai, China) containing 10 × protease inhibitor cocktail (Thermo Fisher Scientific) at 4 °C for 30 min. After centrifugation for 20 min at 12 000 rpm, the supernatants were mixed with 5 × loading buffer. Total protein (20‐30 µg) was separated by 6%−10% sodium dodecyl sulfate‐polyacrylamide gel electrophoresis and transferred to difluoride membrane filter (1 620 177, BIO‐RAD). The blots were blocked in 5% BSA for 1 h, incubated with primary antibodies overnight at 4 °C and secondary antibodies at room temperature for 40 min. GAPDH was used as a loading control. The antibodies for western blot were as follows: FAK antibody (CST, 3285S) (1:1000), FAK antibody (CST, 71433S) (1:1000), rabbit polyclonal to Calpain 2 antibody (Abcam, ab39165) (1:1000), Mouse monoclonal antibody against Flag (Sigma, F1804) (1:1000) and GAPDH antibody (Proteintech, 10494‐1‐AP) (1:1000). The quantification of western blot was conducted by Image‐J (National Institutes of Health).

### RNA Pulldown

Full‐length RNA of FAISL were synthesized by the MaxiScript T7 kit (Ambion), labeled with biotin using Pierce RNA 3′ End Desthiobiotinylation Kit (Thermo Fisher Scientific, 20 163) and purified by Licl solution in PARIS kit (Thermo Fisher Scientific, AM1921). RNA pulldown was performed by Magnetic RNA‐Protein Pull‐Down Kit (Thermo Fisher Scientific, 20 164) according to the manufacturer's manuals. The retrieved proteins were detected by western blot or identified by mass spectrum (MS).

### Preparation of Nanoparticles

The siRNA‐loaded NPs were prepared using a nanoprecipitation method. Briefly, 50 µL of G0‐C14 (5 mg/mL in dimethylformamide) was mixed with 10 µL of siFAISL (0.1 nmol µL^−1^ in water), and then 200 µL of polymer Meo‐PEG‐S‐S‐PLGA solution (20 mg mL^−1^ in dimethylformamide) was added to the mixture. Subsequently, the mixture was added dropwise to 5 mL of deionized water under vigorous stirring (1000 rpm). The formed NP suspension was then purified using an ultrafiltration device (EMD Millipore, MWCO 100 K) and centrifuged to remove the free compounds and organic solvent. After washing with deionized water twice, the obtained siRNA‐loaded NPs (NPs (siCtrl), NPs (siFAISL#1) and NPs (siFAISL#2)) were suspended in deionized water at a siRNA concentration of 1 nmol mL^−1^ for later use.

To determine the encapsulation efficiency (EE%) of siRNA, Cy5‐labeled siFAISL‐loaded NPs were prepared according to the method aforementioned. Subsequently, 5 µL of NP suspension was mixed with 100 µL of DMSO. The standard sample was prepared by mixing 5 µL of naked Cy5‐labeled siFAISL (1 nmol/mL) with 100 µL of DMSO. The fluorescence intensity of Cy5‐labeled siFAISL was measured using a multimode microplate reader (Bio TEK, USA), and the EE% of siFAISL was ≈86%. Size and zeta potential of siRNA‐loaded NPs were examined by dynamic light scattering (DLS, Malvern, USA) and their morphology was viewed under a transmission electron microscope (TEM, FEI, USA). Before observation by TEM, the samples were stained with 1% uranyl acetate and dried under air.

### In Vivo Pharmacokinetics and Biodistribution of Nanoparticles

Following the methods described in the previous section, Cy5‐labeled siRNA encapsulated in nanoparticles was prepared, referred to as siRNA‐loaded NPs. Afterward, we intravenously injected 1 nmol of each siRNA formulation into BALB/C Nude mice (5 weeks old, obtained from Vital River Laboratory). At different time points post‐injection (5 min, 10 min, 30 min, 1 h, 2 h, 4 h, 8 h, 12 h), the Cy5‐siRNA was measured extracted from the orbital blood in nude mice. The fluorescence intensity of the Cy5‐labeled siRNA that was calculated based on a standard curve was measured using a microplate reader (BioTek, USA). For the biodistribution of nanoparticles in nude mice, we intravenously injected 1 nmol of both siRNA formulations into the tumor‐bearing mice with a tumor volume of ≈300 mm^3^. After 24 h, the mice were dissected to gain muscle, heart, liver, spleen, lung, kidney, and tumor tissues, which were measured the fluorescence intensity of Cy5‐siRNA using an IVIS Lumina image system (PerkinElmer, USA).

### In Vitro siRNA Release

The Cy5‐labeled siFAISL‐loaded NPs were suspended in 1 mL of PBS solution (pH 7.4) and then transferred to a Float‐a‐lyzer G2 dialysis device (MWCO 100 kDa, Spectrum) that was immersed in PBS buffer (pH 7.4, with/without GSH) at 37 °C. At a predetermined interval, 5 µL of the NP suspension was withdrawn and mixed with 100 µL of DMSO. The fluorescence intensity of Cy5‐labeled siFAISL was determined using a multimode microplate reader (Bio TEK, USA).

### Animal Experiments

BALB/C nude or NOD/SCID SPF‐level female mice which are 3–5 weeks old and 14–16 g in weight were purchased from Vital River Laboratory and housed in the barrier environment of the Experimental Animal Center, Sun Yat‐sen University. All animal research was carried out in accordance with the protocols approved by the Institutional Animal Care and Use Committee of Sun Yat‐sen Memorial Hospital (Approval Number AP20220005).

To evaluate the effect of siFAISL on the xenograft tumor growth, each nude mouse was subcutaneously injected with 1 × 10^6^ MDA‐MB‐231 cells. When the tumors were palpable, the tumor‐bearing mice were randomly divided into 4 groups (*n* = 8 per group) and subjected to intravenous tail vein injections every other day as follows: i) PBS, ii) NPs (siCtrl), iii) NPs (siFAISL#1), iv) NPs (siFAISL#2), for a total of three treatment cycles. After the start of treatment, the tumor size was measured every 4–5 days, and the tumor volumes were calculated using the formula “volume = length × width × width / 2”. Meanwhile, the weight of the nude mice was also monitored using an electronic balance. At humane endpoint, the tumor tissues were collected, photographed, weighed, and half of the tissues were frozen at −80 °C for subsequent RNA and protein extraction. The other half was fixed with 4% paraformaldehyde, embedded in paraffin, and sectioned for immunohistochemical analysis.

For orthotopic xenograft and metastatic tumor model, 1 × 10^6^ metastatic MDA‐MB‐231‐luciferase cells were orthotopically injected into the mammary fat pad of NOD/SCID mice (*n* = 10 per group) in sterile PBS. Tumor metastasis was monitored by examining the average radiance using bioluminescence imaging. Prior to imaging, D‐luciferin substrate was intraperitoneally injected to mice and the mice were viewed by IVIS Lumina Ill (PerkinElmer, USA) imaging system. At the humane endpoint, mouse tumors were frozen at −80 °C for subsequent RNA and protein extraction, and lungs were harvested, photographed, fixed with 4% paraformaldehyde for immunohistochemical analysis. In the end, survival data was collected and Kaplan‐Meier survival curve was plotted.

### Statistical Analysis

All statistical analysis were performed using GraphPad Prism 8.0 software (California, USA). Comparisons between two groups were assessed by the two‐sided Student's t test. For survival analysis, the Kaplan‐Meier survival curves were plotted using GraphPad Prism. The Mann‐Whitney test and chi‐square (*χ*2) test were used to analyze the relevance of ISH and IHC data with tumor clinicopathological characteristics. Spearman analysis was used to measure the correlation of FAK and FAISL levels. Differences at *p* < 0.05 were regarded as statistically significant.

## Conflict of Interest

The authors declare no conflict of interest.

## Author Contributions

Y.Z., S.W., Z.C., and R.X. contributed equally to this work. E.S. and M.L. designed and supervised the study. Y.Z., S.W., Z.C., S.L., R.W., and Y.Z. performed experiments and acquired data. L.Y. and J.L. analyzed data. Y.Z., R.X., and X.X. designed and evaluated the methodology. Y.Z. and R.W. validated the results of the experiment. Y.Z. and M.L. wrote the paper. E.S. and M.L. provided financial support, resources, and experimental supervision. All authors read and approved the final manuscript.

## Supporting information



Supporting Information

Supporting Information

## Data Availability

The RIP sequencing data are deposited in NCBI Gene Expression Omnibus (GEO) dataset with the accession number GSE268194.

## References

[1] B. Y. Lee , P. Timpson , L. G. Horvath , R. J. Daly , Pharmacol. Therapeut. 2015, 146, 132. 10.1016/j.pharmthera.2014.10.001 25316657

[2] V. M. Golubovskaya , L. Ylagan , A. Miller , M. Hughes , J. Wilson , D. Wang , E. Brese , W. Bshara , S. Edge , C. Morrison , W. G. Cance , BMC Cancer 2014, 14, 769. 25326692 10.1186/1471-2407-14-769 PMC4213510

[3] a) Y. Fan , X. Qu , Y. Ma , Y. Liu , X. Hu , Oncol. Lett. 2016, 12, 1113; 27446403 10.3892/ol.2016.4730 PMC4950184

[4] F. J. Sulzmaier , C. Jean , D. D. Schlaepfer , Nat. Rev. Cancer 2014, 14, 598. 25098269 10.1038/nrc3792 PMC4365862

[5] a) J. C. Dawson , A. Serrels , D. G. Stupack , D. D. Schlaepfer , M. C. Frame , Nat. Rev. Cancer 2021, 21, 313; 33731845 10.1038/s41568-021-00340-6 PMC8276817

[6] X. Sun , H. Gao , Y. Yang , M. He , Y. Wu , Y. Song , Y. Tong , Y. Rao , Signal Transduct. Target Ther. 2019, 4, 64. 31885879 10.1038/s41392-019-0101-6 PMC6927964

[7] L. Statello , C. J. Guo , L. L. Chen , M. Huarte , Nat. Rev. Mol. Cell Biol. 2021, 22, 96. 33353982 10.1038/s41580-020-00315-9 PMC7754182

[8] a) Y. P. Li , F. F. Duan , Y. T. Zhao , K. L. Gu , L. Q. Liao , H. B. Su , J. Hao , K. Zhang , N. Yang , Y. Wang , Nat. Commun. 2019, 10, 1368; 30911006 10.1038/s41467-019-08911-w PMC6433952

[9] a) S. Z. Eslami , L. E. Cortes‐Hernandez , F. Thomas , K. Pantel , C. Alix‐Panabieres , Br. J. Cancer 2022, 127, 800; 35484215 10.1038/s41416-022-01819-1 PMC9427839

[10] a) S. U. Khan , K. Fatima , F. Malik , Clin. Exp. Metastasis 2022, 39, 715; 35829806 10.1007/s10585-022-10172-9

[11] a) S. G. Pollan , P. C. Teng , Y. J. Jan , J. Livingstone , C. Huang , M. Kim , J. Mariscal , M. Rodriguez , J. F. Chen , S. You , D. DiVizio , P. C. Boutros , K. S. Chan , O. Rasorenova , A. Cress , D. Spassov , M. Moasser , E. M. Posadas , S. J. Freedland , M. R. Freeman , J. J. Zheng , B. S. Knudsen , Am. J. Clin. Exp. Urol. 2021, 9, 350; 34541033 PMC8446766

[12] a) H. H. Chuang , Y. Y. Zhen , Y. C. Tsai , C. H. Chuang , M. Hsiao , M. S. Huang , C. J. Yang , Int. J. Mol. Sci. 2022, 23, 1726; 35163650 10.3390/ijms23031726 PMC8836199

[13] A. Li , J. Zhang , Z. Zhou , BMC Bioinformatics 2014, 15, 311. 25239089 10.1186/1471-2105-15-311 PMC4177586

[14] a) K. T. Chan , D. A. Bennin , A. Huttenlocher , J. Biol. Chem. 2010, 285, 11418; 20150423 10.1074/jbc.M109.090746 PMC2857020

[15] P. C. Kerstein , K. M. Patel , T. M. Gomez , J. Neurosci. 2017, 37, 1568. 28069919 10.1523/JNEUROSCI.2769-16.2016 PMC5299572

[16] N. O. Carragher , V. J. Fincham , D. Riley , M. C. Frame , J. Biol. Chem. 2001, 276, 4270. 11069922 10.1074/jbc.M008972200

[17] K. T. Chan , D. A. Bennin , A. Huttenlocher , J. Biol. Chem. 2010, 285, 11418. 20150423 10.1074/jbc.M109.090746 PMC2857020

[18] a) S. Gil‐Parrado , A. Fernandez‐Montalvan , I. Assfalg‐Machleidt , O. Popp , F. Bestvater , A. Holloschi , T. A. Knoch , E. A. Auerswald , K. Welsh , J. C. Reed , H. Fritz , P. Fuentes‐Prior , E. Spiess , G. S. Salvesen , W. Machleidt , J. Biol. Chem. 2002, 277, 27217; 12000759 10.1074/jbc.M202945200

[19] S. Li , L. Xu , G. Wu , Z. Huang , L. Huang , F. Zhang , C. Wei , Q. Shen , R. Li , L. Zhang , X. Xu , Adv. Sci. (Weinh) 2023, 10, e2207118. 37203277 10.1002/advs.202207118 PMC10323624

[20] Y. Kawasaki , M. Komiya , K. Matsumura , L. Negishi , S. Suda , M. Okuno , N. Yokota , T. Osada , T. Nagashima , M. Hiyoshi , M. Okada‐Hatakeyama , J. Kitayama , K. Shirahige , T. Akiyama , Cell Rep. 2016, 16, 2554. 27568568 10.1016/j.celrep.2016.08.015

[21] L. Yang , X. Dong , Z. Liu , J. Tan , X. Huang , T. Wen , H. Qu , Z. Wang , Elife 2022, 11, 1726. 10.7554/eLife.79811 PMC9744440 36458816

[22] a) S. Gu , L. Qian , Y. Liu , J. Miao , H. Shen , S. Zhang , G. Mao , Exp. Ther. Med. 2021, 21, 644; 33968175 10.3892/etm.2021.10076 PMC8097213

[23] a) M. Yang , H. Xiang , G. Luo , Biochem. Pharmacol. 2024, 224, 116246; 38685282 10.1016/j.bcp.2024.116246

[24] P. M. Cromm , K. T. G. Samarasinghe , J. Hines , C. M. Crews , J. Am. Chem. Soc. 2018, 140, 17019. 30444612 10.1021/jacs.8b08008

[25] M. Coan , S. Haefliger , S. Ounzain , R. Johnson , Nat. Rev. Genet. 2024, 578, 6381. 10.1038/s41576-024-00693-2 38424237

[26] R. Cheng , F. Feng , F. Meng , C. Deng , J. Feijen , Z. Zhong , J. Control Release 2011, 152, 2. 21295087 10.1016/j.jconrel.2011.01.030

[27] a) M. Winkle , S. M. El‐Daly , M. Fabbri , G. A. Calin , Nat. Rev. Drug Discov. 2021, 20, 629; 34145432 10.1038/s41573-021-00219-z PMC8212082

[28] a) X. Cao , X. Zhou , F. Hou , Y. E. Huang , M. Yuan , M. Long , S. Chen , W. Lei , J. Zhu , J. Chen , T. Zhang , A. Y. Guo , W. Jiang , Nucleic Acids Res. 2024, 52, D1393; 37953323 10.1093/nar/gkad1042 PMC10767907

