# Supplementary material for: LncRNA FAISL Inhibits Calpain 2‐Mediated Proteolysis of FAK to Promote Progression and Metastasis of Triple Negative Breast Cancer
Source: Adv Sci (Weinh). 2024 Sep 17;11(42):2407493. doi: 10.1002/advs.202407493 (PMC11558121; doi:10.1002/advs.202407493)
Supplement: Supplementary file 1 — Supporting Information [file ADVS-11-2407493-s002.docx]

**Supplementary Materials**

**LncRNA FAISL inhibits Calpain 2-mediated** **proteolysis of FAK to promote progression and metastasis of triple negative breast cancer**

*Yunmei Zhang*^#^, *Shiyu Wei*^#^, *Zhengjie Chen*^#^, *Rui Xu*^#^, *Shu-Rong Li*, *Lili You*, *Ruiyue Wu*, *Yin Zhang, Jian-You Liao, Xiaoding Xu, Erwei Song*, Man-Li Luo**

^#^ These authors contributed equally to the manuscript.

* Correspondence to Erwei Song ([songew@mail.sysu.edu.cn](mailto:songew@mail.sysu.edu.cn)) and Man-Li Luo ([luomli@mail.sysu.edu.cn](mailto:luomli@mail.sysu.edu.cn))

**
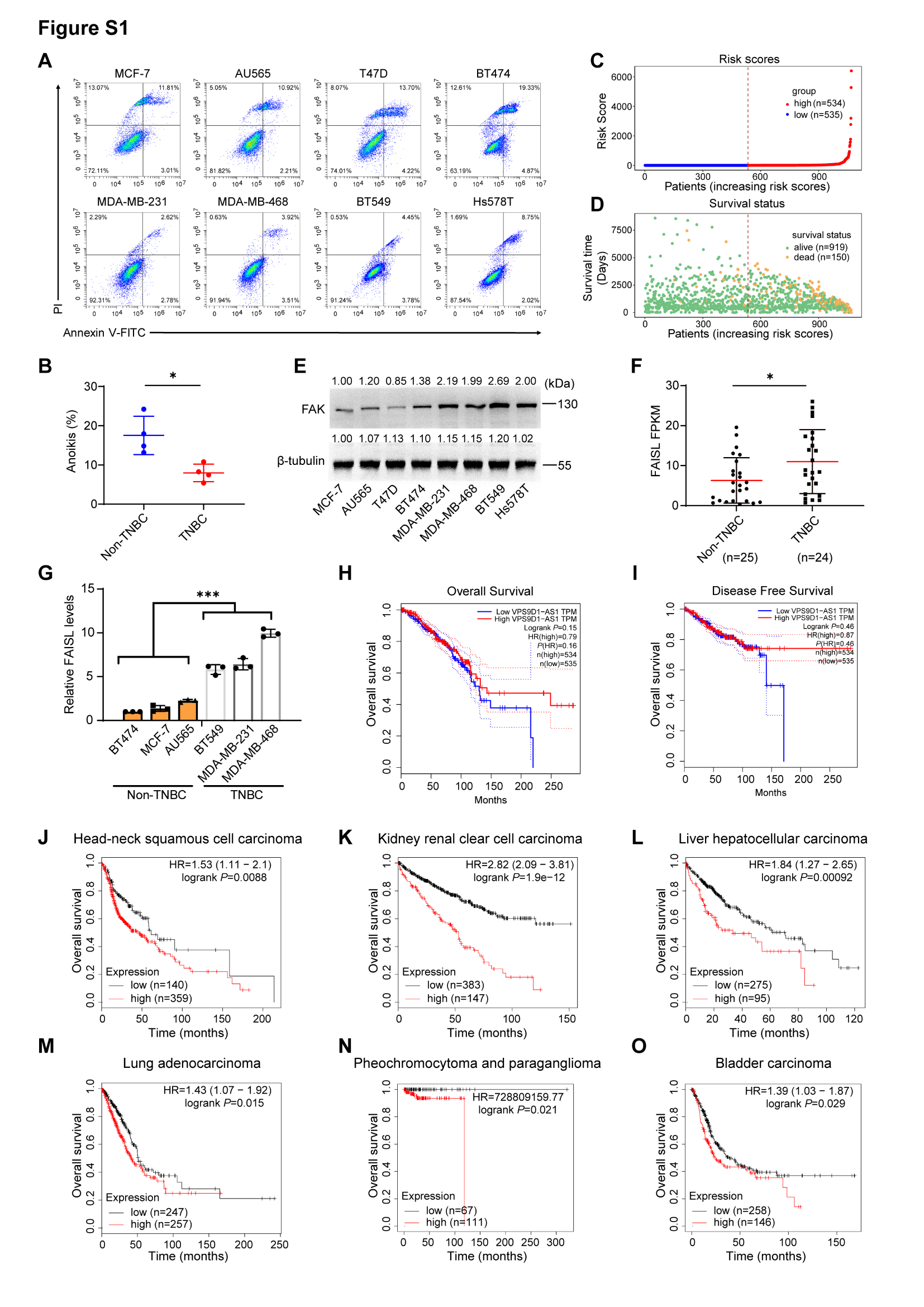
**

**Figure S1. FAISL is a FAK-associated lncRNA and correlates with poor prognosis in pan cancer patients.**

**A.** Flow cytometry assay showing the detachment-induced anoikis rate in non-TNBC and TNBC cell lines.

**B.** The quantification of the anoikis rate of cells in **(A)**.

**C.** The division of TCGA BRCA patients into high- and low-risk group by the median cut-off of the risk scores.

**D.** The survival time and survival status of TCGA BRCA patients with different risk scores of FA pathways related genes.

**E.** Western blot of FAK protein in non-TNBC and TNBC cell lines. The relative intensities were indicated above the bands.

**F.** The expression of FAISL in non-TNBC and TNBC cell lines from Broad Institute Cancer Cell Line Encyclopedia.

**G.** RT-qPCR of FAISL in non-TNBC and TNBC cell lines.

**H-I.** Association of FAISL expression with overall survival **(H)** and disease-free survival **(I)** in TCGA breast cancer dataset.

**J-O.** Kaplan-Meier analysis of FAISL expression and overall survival of head-neck squamous cell carcinoma **(J)**, kidney renal clear cell carcinoma **(K)**, liver hepatocellular carcinoma **(L)**, lung adenocarcinoma **(M)**, pheochromocytoma and paraganglioma **(N)** and bladder Carcinoma **(O)** in the KM Plotter RNA-seq datasets.

Data are presented as mean ± SD of experimental triplicates **(G)**. *P*-values were assessed with two-tailed Student’s *t*-test **(B, F-G)**, with log-rank test **(H-O)**. (**P* < 0.05, ***P* < 0.01, ****P* < 0.001.)

**
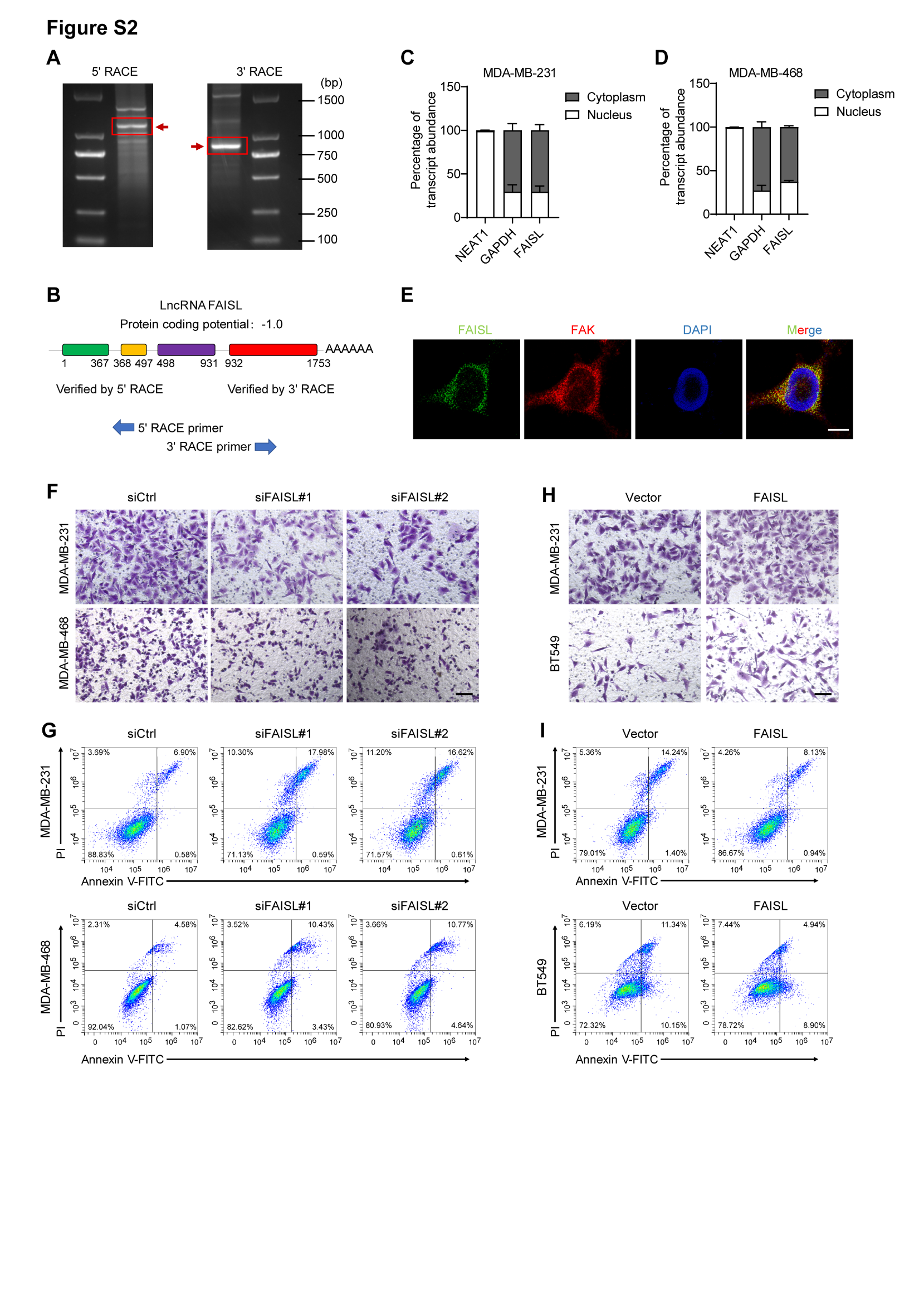
**

**Figure S2. Characterization of FAISL in TNBC cells.**

**A.** Agarose gel showing 5’ and 3’ RACE products of FAISL.

**B.** Schematic diagram of FAISL sequence by 5’ and 3’ RACE.

**C-D.** RT-qPCR analysis of FAISL in the cytoplasm and nuclei fractionations of MDA-MB-231 **(C)** and MDA-MB-468 cells **(D)**.

**E.** FISH and IF images showing the co-localization of lncRNA FAISL (green) and FAK protein (red) in MDA-MB-231. Scale bar, 10 µm.

**F.** Representative images of the transwell migration assay after knocking down FAISL. Scale bar, 50 µm.

**G.** Flow cytometry showing the detachment-induced anoikis rate of cells after knocking down FAISL. Cells were cultured in suspension for 12 hours.

**H.** Representative images of the transwell migration assay after overexpressing FAISL. Scale bar, 50 µm.

**I.** Flow cytometry showing the detachment-induced anoikis rate of cells after overexpressing FAISL. Cells were cultured in suspension for 24 hours.

Data are presented as mean ± SD of experimental triplicates **(C-D)**.

**
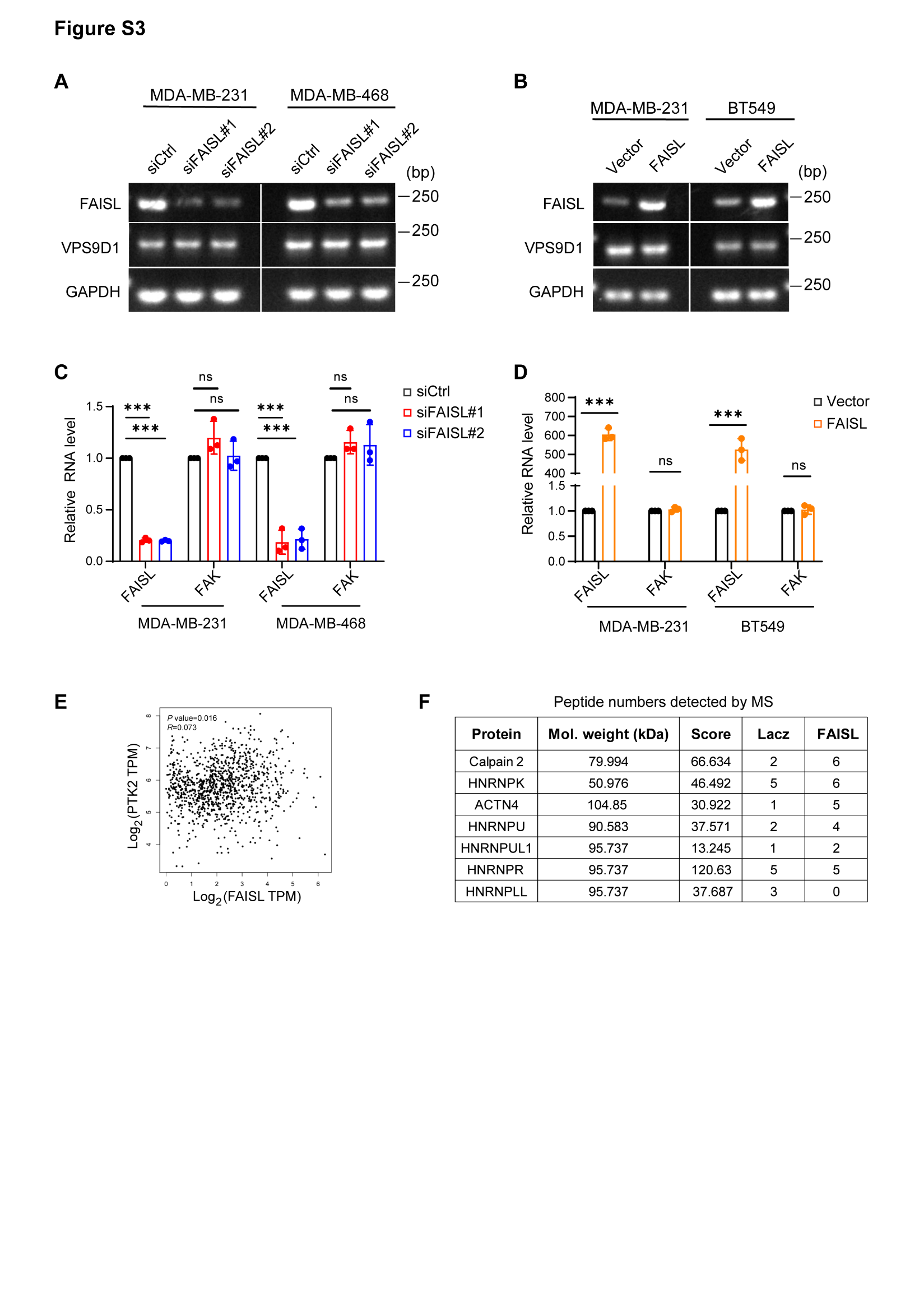
**

**Figure S3.** **The knockdown and overexpression of FAISL do not affect FAK mRNA level in TNBC cells**

**A-B.** Silencing **(A)** or overexpressing **(B)** FAISL in TNBC cells did not affect the expression of VPS9D1 mRNA.

**C-D.** Silencing **(C)** or overexpressing **(D)** FAISL in TNBC cells did not affect the expression of FAK mRNA.

**E.** FAISL expression is not associated with FAK mRNA levels in TCGA breast cancer database.

**F.** The matched peptide numbers identified by mass spectrometry.

Data are presented as mean ± SD of experimental triplicates **(C-D)**. *P*-values were assessed with two-tailed Student’s *t*-test **(C-D)** or Spearman analysis **(E)**. (ns means not significant, **P* < 0.05, ***P* < 0.01, ****P* < 0.001.)

**
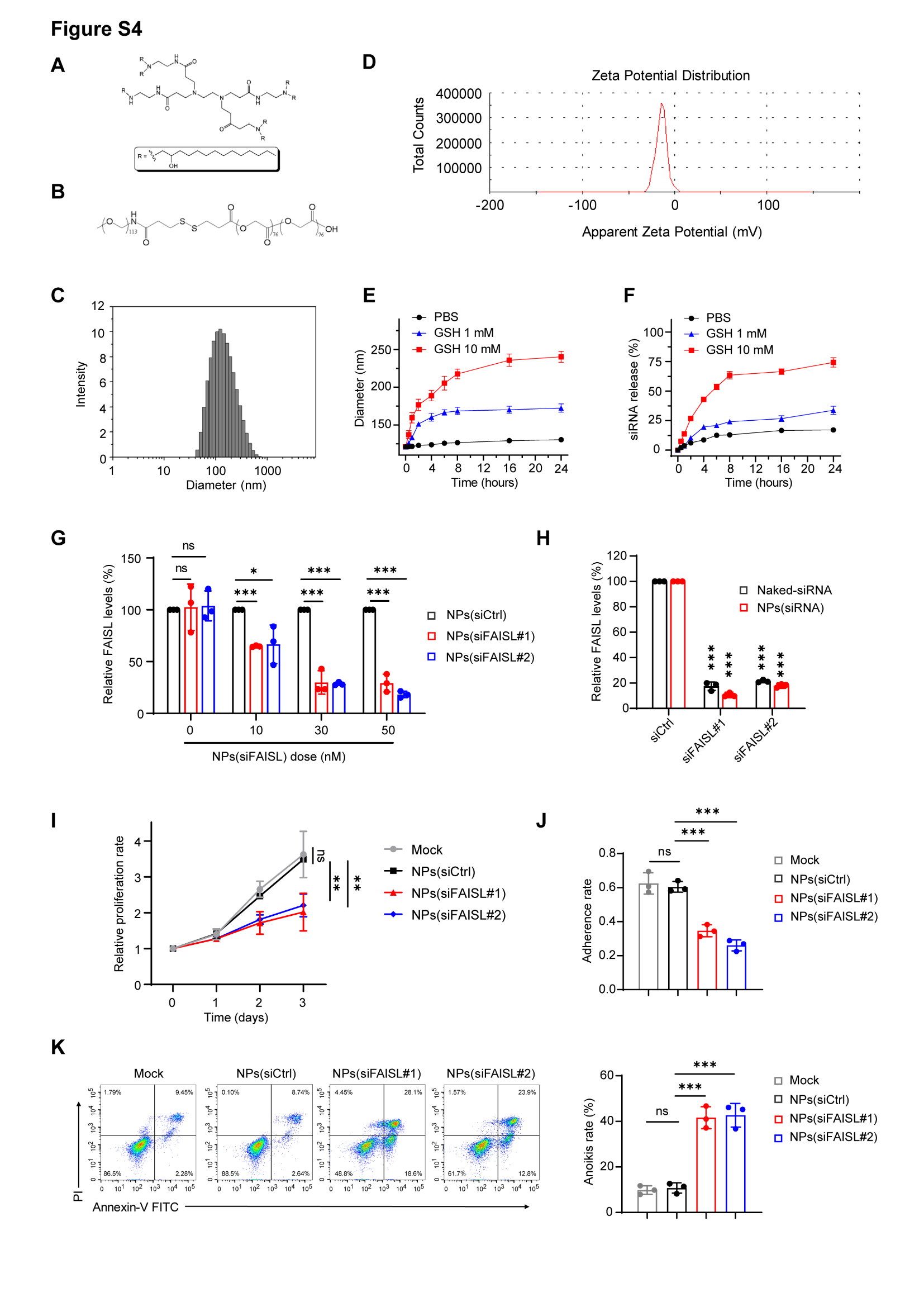
**

**Figure S4. Reduction-responsive NPs-mediated FAISL silencing inhibits TNBC cell adhesion and anoikis resistance.**

**A.** Chemical structure of the amphiphilic cationic lipid G0-C14.

**B.** Chemical structure of the meo-PEG-S-S-PLGA.

**C.** The diameter of the NPs (siRNA) dispersed in aqueous solution.

**D.** Apparent zeta potential distribution of the NPs (siRNA) dispersed in aqueous solution.

**E-F.** Cumulative siRNA release **(F)** and diameter **(E)** of the NPs (Cy5-siRNA) incubated in the PBS solution with different concentration of GSH.

**G.** RT-qPCR showing the knockdown efficiency of FAISL in MDA-MB-231 cells treated with NPs (siCtrl/siFAISL) at different siRNA doses.

**H.** RT-qPCR showing the knockdown efficiency of FAISL in MDA-MB-231 cells treated with naked siRNA containing transfection reagent or NPs (siCtrl/siFAISL).

**I-K.** The proliferation rate **(I)**, adhesion assay **(J)** and anoikis analysis **(K)** of MDA-MB-231 cells treated with NPs (siCtrl) or NPs (siFAISL) at a dose of 50 nM.

Data are presented as mean ± SD of experimental triplicates **(E-K)**. *P*-values were assessed with two-tailed Student’s *t*-test **(E-K)**. (ns means not significant, **P* < 0.05, ***P* < 0.01, ****P* < 0.001.)

**
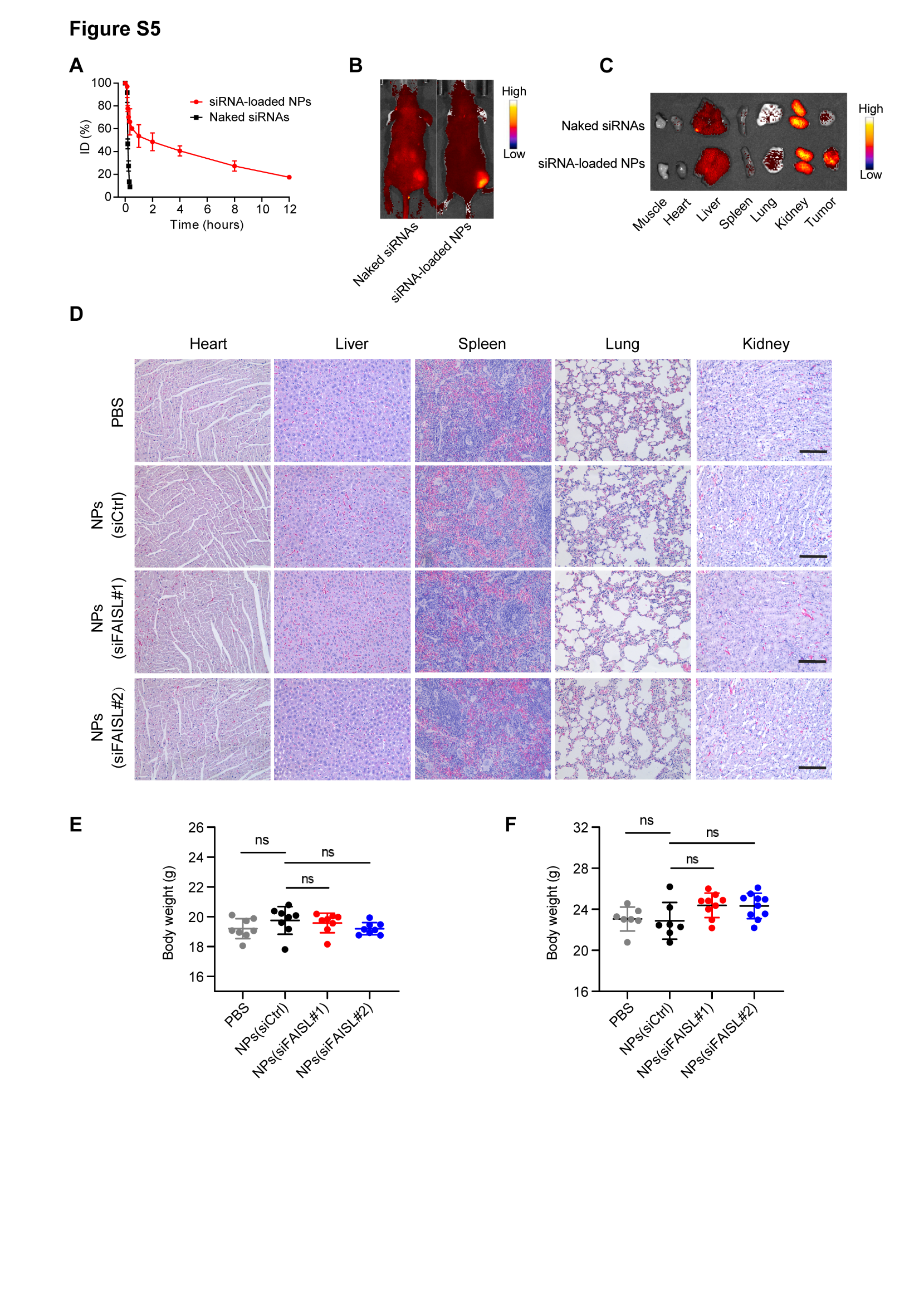
**

**Figure S5. Pharmacokinetics and biodistribution of the siRNA loaded NPs in mice.**

**A.** Blood circulation of naked Cy5-labeled siFAISL and the Cy5-labeled siFAISL loaded NPs. SiFAISL loaded NPs referred to the use of residual Cy5-labeled siFAISL in the blood as the detection index.

**B.** Overlaid fluorescence image of the MDA-MB-231 tumor-bearing nude mice at 24 hours post injection of the Cy5-labeled siFAISL loaded NPs or naked Cy5-labeld siFAISL.

**C.** Representative fluorescent images of major organs and tumors from tumor-bearing nude mice euthanized at 24 hours after injection of Cy5-labeled naked siRNA or siRNA-loaded nanoparticles.

**D.** HE staining of histological sections of the major organs through three consecutive treatment of PBS, NPs (siCtrl), NPs (siFAISL#1) and NPs (siFAISL#2). Scale bar, 500 µm.

**E-F.** Body weights of the MDA-MB-231 xenograft tumor-bearing nude mice **(E)** or Luc-MDA-MB-231 metastatic tumor-bearing NOD/SCID mice **(F)** treated with siRNA at a 1 nmol dose per mouse.

For **A-D**, n = 3 mice per group. Each dot represents one mouse **(E-F)**. For **A, E-F**, mean ± SD are shown, and *P*-values were determined using two-tailed Student’s *t*-test **(A, E-F)**. (ns means not significant.)

**Table S1. siRNA/shRNA and probe sequences**.

| **Gene name** | **Sequence** |
| --- | --- |
| **siRNAs and shRNAs target sequences** | |
| siFAISL#1 | CTTGGCATGGAGCACCTCT |
| siFAISL#2 | GGGATCGCCTCACCCATCT |
| shFAISL#1 | CTTGGCATGGAGCACCTCT |
| shFAISL#2 | GGGATCGCCTCACCCATCT |
| **Probes used in ISH assays** | |
| FAISL | TATCTCAGCAGTAAGTAACAGT |
| **Probes used in FISH assays (mixed probe)** | |
| FAISL#1 | GAGTACGAACAGGACCATGT |
| FAISL#2 | CAGGAGGTTTTGCAGCCAAG |
| FAISL#3 | AATCCGGGAGGATGAGAGAG |
| FAISL#4 | AGATCTTCCAGAAGCTTCAG |
| FAISL#5 | GAAAGCTCTCTCCTTTTCTG |
| FAISL#6 | CCGTGTATACTCCGATGAAG |
| FAISL#7 | CATGGCTTGCAGGGAAAACA |
| FAISL#8 | GACACCTCCAAGATGCTGAA |
| FAISL#9 | AAGTAACAGTGGTAGAGCCG |
| FAISL#10 | GTTGCCTCATTTTATCTCAG |
| FAISL#11 | TCTCTGTAAAACGGCCACTT |
| FAISL#12 | CATGCCAAGCTACGGGAAGG |
| FAISL#13 | CAGAAGGGCTGTGTGAGCAG |
| FAISL#14 | GAGTCACAGAGAGGCAGCTC |
| FAISL#15 | GAAGTTTTCTGTGAAGGGCA |
| FAISL#16 | CGGGGCGGGATGAGAAAATG |
| FAISL#17 | CAAGGTGGGGACGGGTAAGG |
| FAISL#18 | GCTCCAAGATTGAGGAGTTG |
| FAISL#19 | TCAGGGGGAGCTAACAGTTG |
| FAISL#20 | ACTGGACGTAGGATCACACA |
| FAISL#21 | CTGGACGTGGGATCACAGAG |
| FAISL#22 | CTGGACGTGGGATCACAGAG |
| FAISL#23 | CTGGACGTGGGAGCACAGAG |
| FAISL#24 | CTGGACGTGGGAGCACAGAG |
| FAISL#25 | CTGGACGTGGGAGCACAGAG |
| FAISL#26 | CTGGACGTGGGATCACAGAG |
| FAISL#27 | CTGGACATAGAGCACAGAGG |
| FAISL#28 | GCTGCACAAAGGATCACAGA |
| FAISL#29 | AGTTCCGCAATGAACACATT |

**Table S2. Primers for RT-qPCR.**

| Gene name | Primer | Sequence |
| --- | --- | --- |
| FAISL | sense | ATGGGTAACCAGGGGTCAAG |
|  | antisense | AGTAACAGTGGTAGAGCCGA |
| PICSAR | sense | AGGGTCAAGCAGAACTGTGG |
|  | antisense | TGCATTGCTTAGGTGGGGAG |
| Lnc-SPATA21-4 | sense | CAGGTCACTTTGCATCTGCT |
|  | antisense | GGACAAGGTGCTCCACTAGAA |
| LINC01137 | sense | TGAAAGCTCACCGGTCCTTG |
|  | antisense | TTCTGCGGGGACAACATCTG |
| Lnc-ALG1L-2:2 | sense | TCAAGCAGCAACCCTAGGAC |
|  | antisense | GGGTGTGTTGCAGACAGTCA |
| Lnc-GOLGA6L6-10 | sense | GGTCCCGATGGGTTCTCATT |
|  | antisense | GATGCTGCTGGAAGTCACCT |
| Lnc-PILRB-1 | sense | GCCTCTCATTCCCTGTCTGTG |
|  | antisense | ATTTGGAGGTGACCGGGAGG |
| MMP24-AS1:3 | sense | GGACTTCTTTCCAGTCCCCG |
|  | antisense | GTCCCTGCGTCTCTTACACC |
| SNHG7:1 | sense | CTCTGCTCCGCAATGTGTGA |
|  | antisense | CCGCGTGGCTGTTTTTGAAC |
| Lnc-AGRP-1:4 | sense | ATCCTTTCCACCAACCAGCC |
|  | antisense | CACGCAGACCCTAAAACGGT |
| VPS9D1 | sense | CCCCAGAAGAAGCTGGAGTG |
|  | antisense | CACCACGAAGGACAGGATGG |
| FAK | sense | ACATTATTGGCCACTGTGGATGAG |
|  | antisense | GGCCAGTTTCATCTTGTTGATGAG |
| GAPDH | sense | CTCCTCCACCTTTGACGCTG |
|  | antisense | TCCTCTTGTGCTCTTGCTGG |

**Table S3. Primers for RACE.**

| Gene name | 5’/3’ Primer | Sequence |
| --- | --- | --- |
| FAISL | 5’ RACE primer | GATTACGCCAAGCTTGGGTCCAGGGGCGAGTCACAGAGAGG |
|  | 3’ RACE primer | GATTACGCCAAGCTTCCTCACCCATCTCCCACCGGCTGTGTCC |

**Table S4. Correlation of FAISL expression with the clinicopathological status in breast cancer tissues.**

|  | | **No. of patients** | **FAISL expression^#^** | | ***P*-values** |
| --- | --- | --- | --- | --- | --- |
|  |  | **low** | **high** |  | |
| **Tumor Size (cm)** | |  |  |  | |
| ＜2 | 56 | 37 (66.1%) | 19 (33.9%) | 0.0489 | |
| ≥2 | 110 | 55(50%) | 55 (50%) |  | |
| **Lymph Node Metastasis** | | |  |  | |
| Negative | 141 | 89 (63.1%) | 52 (36.9%) | ＜0.0001 | |
| Positive | 25 | 22 (88%) | 3 (12%) |  | |
| **Tumor metastasis** | | | | | |
| Negative | 146 | 87 (59.6%) | 59 (40.4%) | 0.0035 | |
| Positive | 20 | 15 (75%) | 5 (25%) |  | |
| **Ki67 (% of tumor cells)** | | | | | |
| ＜14% | 24 | 20 (83.3%) | 4 (16.7%) | 0.0029 | |
| ≥14% | 142 | 72 (50.7%) | 70 (49.3%) |  | |

^#^FAISL expression in breast cancer tissue was determined by ISH.
